# A Secretary Bird Optimizer with Good Point Set Initialization and Trend-Aware Search for Benchmark and Portfolio Optimization

**DOI:** 10.3390/biomimetics11100762

**Published:** 2026-10-21

**Authors:** Zhe Ma, Jian Hai, Ming Yang

**Affiliations:** 1 School of Economics, Management and Law, Jilin Normal University, Siping 136000, China; mazhe@jlnu.edu.cn; 2 Department of Business Administration, Sejong University, Seoul 05006, Republic of Korea; 26150021@sju.ac.kr

**Keywords:** secretary bird optimization algorithm, biomimetic optimization, GT-SBOA, global optimization, CEC2017, good point set, trend-aware search, portfolio optimization case study

## Abstract

Accepted population displacements provide directional information that can be reused in subsequent derivative-free search. This paper develops the Good Point Set and Trend-Aware Secretary Bird Optimization Algorithm (GT-SBOA), combining a dimension-admissible number-theoretic initialization with an auxiliary search direction formed from individual and population-center motion. The auxiliary update is applied to the worse-performing half of the population in every complete sweep, with all objective calls charged to a common evaluation budget. Evaluation covers 29 CEC2017 functions, a two-component ablation, directional-weight sensitivity, and five OR-Library portfolio instances. In the comparison at D=30, GT-SBOA has a mean rank of 2.4138 across eleven algorithms, with 14 lowest mean errors and a significant win/tie/loss record of 16/4/9 against SBOA. The four-method comparison places it first at D=50 and third at D=100, showing that the aggregate advantage is dimension-dependent. The portfolio evaluation retains native initialization in the complete-method comparison and uses GD and IGD to assess frontier accuracy and coverage, with a common-initialization control against the parent algorithm. These comparisons delimit the conditions under which displacement-guided updates offer useful search information within a fixed evaluation budget.

## 1. Introduction

Population-based optimization is useful when candidate solutions can be evaluated but reliable derivative information is unavailable or costly [1,2,3]. Its practical challenge is to use a finite evaluation budget effectively across exploration and refinement. Initial population placement determines the information available at the start of a run, while the update rule determines how that information is converted into subsequent trials. Improving either component therefore requires assessing solution quality together with the number of objective calls.

Evolutionary and swarm-based methods use different forms of population information. Differential Evolution (DE) constructs trial directions from differences between current solutions [4], whereas Particle Swarm Optimization (PSO) combines velocity with individual and social memories [5]. Recent search methods also employ physical, behavioral and learning-based update rules, including RIME [6], the Educational Competition Optimizer (ECO) [7], the Parrot Optimizer (PO) [8], and Status-based Optimization (SBO) [9]. These designs differ in the information they preserve and the evaluation cost of applying it.

The Secretary Bird Optimization Algorithm (SBOA) [10] combines hunting and escape updates inspired by the secretary bird, *Sagittarius serpentarius*. Hunting progresses through differential exploration, Brownian movement and Lévy-flight refinement; escape updates provide additional candidate directions. Greedy selection retains improvements. The resulting search uses current positions and elite information, but accepted displacements are not explicitly retained as a directional signal. In addition, independently sampled initial populations can have uneven finite-sample coverage.

SBOA variants address different parts of this search process [11]. UTFSBOA combines directional search, an energy-related update and Cauchy–Gaussian crossover [12]. ISBOA couples chaotic/opposition initialization with differential variation and adaptive escape control [13]. The present work considers a complementary information source: the movement that survives greedy selection in the preceding hunting–escape sweep. An individual’s accepted displacement describes its local progress, while the centroid displacement summarizes collective movement. Combining these signals offers a compact way to guide additional trials, but those trials also consume evaluations that could otherwise support the parent operators.

GT-SBOA combines a good-point construction [14] with this displacement-guided update. The initialization uses a dimension-dependent prime that avoids repeated cosine coordinates. During search, a fixed fraction of the population receives additional candidates built from individual and centroid motion, with perturbations decreasing as the evaluation budget is consumed. The auxiliary operator is applied in every complete sweep; it does not rely on a stagnation detector.

The study makes three contributions. First, it specifies the initialization, displacement timing, selection order and evaluation accounting of GT-SBOA in a reproducible algorithmic form. Second, it compares the complete method with classical and recent optimizers on CEC2017, including UTFSBOA and ISBOA, and separates the two components through a common-data ablation. Third, it evaluates the complete algorithms on portfolio instances, using GD, IGD and matched-return risk, and it includes a common-initialization control against the parent algorithm. The numerical analysis reports function-level differences and dimension-specific rankings alongside aggregate performance.

Section 2 describes SBOA. Section 3 presents GT-SBOA and its computational cost. Section 4 gives the benchmark protocol and comparisons. Section 5 considers portfolio optimization, and Section 6 concludes the study.

## 2. Original Secretary Bird Optimization Algorithm

The Secretary Bird Optimization Algorithm (SBOA) [10] is a swarm-intelligence metaheuristic inspired by the foraging, hunting, and predator-avoidance behaviors of secretary birds (*Sagittarius serpentarius*). SBOA coordinates a population of *N* agents across a *D*-dimensional search space using a two-stage iterative cycle: a hunting phase for global exploration and an escape phase for local exploitation.

### 2.1. Mathematical Model of SBOA

The initial positions of the *N* candidate solutions are sampled uniformly across the decision space bounded by $\left[lb_{j},ub_{j}\right]$:

(1) $$x_{i,j}=lb_{j}+r\times \left(ub_{j}-lb_{j}\right),\;i=1,\ldots ,N,\;j=1,\ldots ,D,$$

where r∼U(0,1). The population matrix X and corresponding objective fitness vector F are formulated as:

(2) $$X={\left[\begin{array}{cccc} x_{1,1} & x_{1,2} & \ldots & x_{1,D}\\ x_{2,1} & x_{2,2} & \ldots & x_{2,D}\\ \vdots & \vdots & \ddots & \vdots \\ x_{N,1} & x_{N,2} & \ldots & x_{N,D} \end{array}\right]}_{N\times D},\;F={\left[\begin{array}{c} F(x_{1})\\ \vdots \\ F(x_{N}) \end{array}\right]}_{N\times 1}.$$

Each optimization iteration executes two sequential behavioral stages—the hunting phase and the escape phase—regulated by greedy selection.

In the hunting phase, update trajectories are partitioned into three time-dependent sub-phases based on the elapsed iteration fraction t/T, where *t* denotes the current iteration, and *T* is the maximum iteration budget. When $t<\frac{1}{3}T$, SBOA employs differential vector perturbations between randomly chosen individuals to foster population diversity:

(3) $$X_{i,j}^{\mathrm{new},P1}=X_{i,j}+\left(X_{{\mathrm{random}}_{1}}-X_{{\mathrm{random}}_{2}}\right)\times R_{1},$$

where $R_{1}$ is a 1×D uniform random vector in ${\left[0,1\right]}^{D}$, and $X_{{\mathrm{random}}_{1}},X_{{\mathrm{random}}_{2}}$ denote two distinct individuals randomly drawn from the swarm.

During the intermediate stage ($\frac{1}{3}T\leq t<\frac{2}{3}T$), search moves toward the elite position guided by Brownian motion:

(4) RB=randn(1,D),

(5) $$X_{i,j}^{\mathrm{new},P1}=X_{\mathrm{best}}+\exp\;\left({\left(t/T\right)}^{4}\right)\times \left(RB-0.5\right)\times \left(X_{\mathrm{best}}-X_{i,j}\right),$$

where RB represents standard Gaussian noise, and $X_{\mathrm{best}}$ is the current global best position.

In the late hunting stage ($t\geq \frac{2}{3}T$), SBOA uses a Lévy flight step (RL) combined with a nonlinear time decay factor to intensify local exploitation around the global best solution:

(6) $$X_{i,j}^{\mathrm{new},P1}=X_{\mathrm{best}}+{\left(1-\frac{t}{T}\right)}^{\left(2\frac{t}{T}\right)}\times X_{i,j}\times RL,$$

with RL defined as:

(7) RL=0.5×Levy(D),

where the Lévy distribution vector is evaluated via Mantegna’s algorithm:

(8) $$\mathrm{Levy}\left(D\right)=\frac{s\times u\times \sigma }{{\left|v\right|}^{1/\eta }},\;\sigma ={\left(\frac{\Gamma (1+\eta )\sin(\pi \eta /2)}{\Gamma \;\left(\frac{1+\eta }{2}\right)\eta \;2^{(\eta -1)/2}}\right)}^{\;1/\eta },$$

with s=1, η=1.5, and independent standard Gaussian vectors u,v∼N(0,I), matching the original Mantegna implementation.

At the end of the hunting phase, greedy selection compares the candidate position $X_{i}^{\mathrm{new},P1}$ against the current position $X_{i}$:

(9) $$X_{i}=\left\{\begin{array}{cc} X_{i}^{\mathrm{new},P1}, & \mathrm{if}\;F\left(X_{i}^{\mathrm{new},P1}\right)<F\left(X_{i}\right),\\ X_{i}, & \mathrm{otherwise}. \end{array}\right.$$

In the subsequent escape phase, SBOA probabilistically switches between two predator-evasion mechanisms:

(10) $$X_{i,j}^{\mathrm{new},P2}=\left\{\begin{array}{cc} X_{\mathrm{best}}+\left(2\times RB-1\right)\times {\left(1-\frac{t}{T}\right)}^{2}\times X_{i,j}, & \mathrm{if}\;\mathrm{rand}<0.5,\\ X_{i,j}+R_{2}\times \left(X_{\mathrm{random}}-K\times X_{i,j}\right), & \mathrm{otherwise}, \end{array}\right.$$

where RB is standard Brownian Gaussian noise, $R_{2}∼U{\left(0,1\right)}^{D}$, $X_{\mathrm{random}}$ is an agent selected randomly from the population, and K=round(1+rand). Greedy selection is reapplied:

(11) $$X_{i}=\left\{\begin{array}{cc} X_{i}^{\mathrm{new},P2}, & \mathrm{if}\;F\left(X_{i}^{\mathrm{new},P2}\right)<F\left(X_{i}\right),\\ X_{i}, & \mathrm{otherwise}. \end{array}\right.$$

### 2.2. Integrated Iteration Workflow

**Figure 1:**
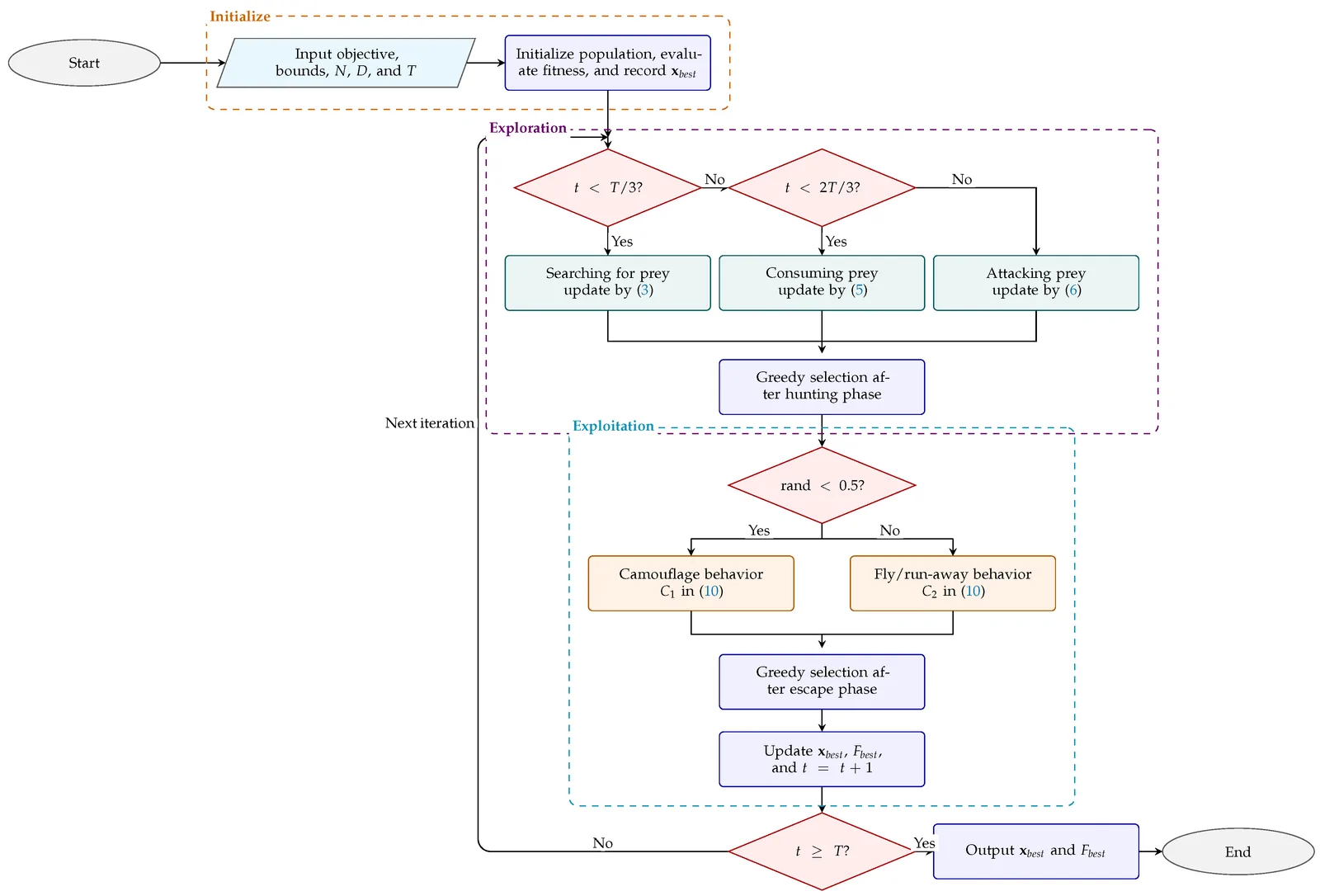
Flowchart of the original Secretary Bird Optimization Algorithm (SBOA).

Following initialization in (1), SBOA evaluates the population and tracks the elite candidate. Across iterations t=1,…,T, individuals sequentially undergo the three-stage hunting update ((3), (5), or (6)) and the dual-mode escape update (10). Elite records are updated whenever an improved fitness value is encountered. Figure 1 illustrates the computational flow of standard SBOA, and Algorithm 1 provides its formal pseudocode.
**Algorithm 1** Original Secretary Bird Optimization Algorithm (SBOA)1:**Input:** Population size *N*, maximum iterations *T*, dimension *D*, bounds lb,ub.2:Initialize population X using (1) and evaluate fitness F.3:Determine $X_{\mathrm{best}}$ and $F_{\mathrm{best}}$.4:**for** t=1 to *T* **do**5:    **for** i=1 to *N* **do**6:        **if** $t<\frac{1}{3}T$ **then**7:           Compute $X_{i}^{\mathrm{new},P1}$ via (3).8:        **else if** $t<\frac{2}{3}T$ **then**9:           Compute RB=randn(1,D).10:           Compute $X_{i}^{\mathrm{new},P1}$ via (5).11:        **else**12:           Draw independent Gaussian u,v and compute σ and RL using (8).13:           Compute $X_{i}^{\mathrm{new},P1}$ via (6).14:        **end if**15:        Repair bounds; evaluate $X_{i}^{\mathrm{new},P1}$ and retain it if fitness improves.16:    **end for**17:    **for** i=1 to *N* **do**18:        Generate RB, $R_{2}$, *K*, and rand.19:        Compute $X_{i}^{\mathrm{new},P2}$ via (10).20:        Repair bounds; evaluate $X_{i}^{\mathrm{new},P2}$ and retain it if fitness improves.21:    **end for**22:    Update $X_{\mathrm{best}}$ if a better solution is found.23:**end for**24:**Output:** Global best solution $X_{\mathrm{best}}$ and $F_{\mathrm{best}}$.

## 3. The Proposed GT-SBOA

GT-SBOA retains the hunting–escape sequence and adds two components: a deterministic initial point set for box-constrained search and an auxiliary update based on accepted displacements. The two components are separately enabled in the ablation. Their evaluations, including unsuccessful candidates, are counted within the same budget as those of the parent algorithm.

### 3.1. Good Point Set Initialization

Following the cosine construction associated with Hua and Wang [14], choose

(12) $$p=\min\left\{q:q\;\mathrm{is}\;\mathrm{prime},\;q\geq 2D+3\right\},\;r_{j}=2\cos\left(\frac{2\pi j}{p}\right),\;j=1,\ldots ,D.$$

The normalized coordinates and their box mapping are

(13) $$g_{i,j}=\left\{ir_{j}\right\},\;i=1,\ldots ,N,\;j=1,\ldots ,D,$$

(14) $$x_{i,j}^{\left(0\right)}=lb_{j}+\left(ub_{j}-lb_{j}\right)g_{i,j},$$

where {a}=a−⌊a⌋. The dimension condition places all angles 2πj/p strictly inside (0,π), where cosine is strictly decreasing; consequently, the generator contains no pair $r_{j}=r_{k}$ with j≠k. In particular, the symmetry j+k=p cannot occur because j+k≤2D<p. No additional prime multiplier is applied. The selected primes are 67, 103 and 211 for D=30, 50 and 100, respectively. Table 1 reports direct coordinate checks.

The same deterministic point set starts each box-constrained run, while subsequent random updates use independent streams. Distinct generating coefficients remove the coordinate-identification defect. For the main portfolio comparison, the good points are mapped to feasible weights by the same normalization repair used for every method. A common-initialization control against SBOA in the portfolio evaluation (Section 5) disables GPS to isolate the search updates.

### 3.2. Trend-Aware Search Trajectory

Let $x_{i}^{\left(t\right)}$ be the position at the start of sweep *t*, and let ${\tilde{x}}_{i}^{\left(t\right)}$ denote its position after the hunting and escape updates have been repaired, evaluated and greedily selected. The accepted individual displacement is

(15) $$\Delta_{i}^{\left(t\right)}={\tilde{x}}_{i}^{\left(t\right)}-x_{i}^{\left(t\right)}.$$

The centroid displacement and composite search direction are then

(16) $$g^{\left(t\right)}=\frac{1}{N}\sum_{i=1}^{N}\Delta_{i}^{\left(t\right)},$$

(17) $$\tau_{i}^{\left(t\right)}=\alpha \Delta_{i}^{\left(t\right)}+\left(1-\alpha \right)g^{\left(t\right)},\;\alpha =0.65.$$

Here, α controls the relative contribution of local and population motion. The coordinate-wise sample standard deviation of the *N* displacement vectors is denoted by $s^{\left(t\right)}$. These quantities are computed once after the parent updates and held fixed during the auxiliary stage, avoiding dependence on the order in which auxiliary candidates are accepted.

Let $n_{t}$ be the number of objective calls consumed at the start of sweep *t* and *B* the common evaluation budget. The normalized progress is

(18) $$\rho_{t}=\min\left(1,n_{t}/B\right).$$

The parent phase switches and decay schedules use $\rho_{t}$ in place of t/T. This defines the search schedule even when extra evaluations change the number of completed sweeps. The m=⌊N/2⌋ individuals with the largest fitness values after the parent updates form the auxiliary target set, with equal fitness resolved by stable individual index. For each target, the primary candidate is

(19) $$\begin{array}{cc} y_{i}^{\left(t\right)}= & {\tilde{x}}_{i}^{\left(t\right)}+\left[0.70(1-\rho_{t})+0.05\right]\tau_{i}^{\left(t\right)}\\ \; & +0.05\left(1-\rho_{t}\right)s^{\left(t\right)}⊙\epsilon_{i}+0.18\left(1-\rho_{t}\right)u_{i}\left({\tilde{x}}_{\mathrm{best}}^{\left(t\right)}-{\tilde{x}}_{i}^{\left(t\right)}\right), \end{array}$$

where $\epsilon_{i}∼N\left(0,I\right)$, $u_{i}∼U\left(0,1\right)$, and ⊙ is the element-wise product. The frozen elite ${\tilde{x}}_{\mathrm{best}}^{\left(t\right)}$ is the best point after the parent updates. With probability $p_{\mathrm{alt}}=0.35$, the primary candidate is replaced by

(20) $${\hat{y}}_{i}^{\left(t\right)}=\left(0.65+0.25u_{1}\right){\tilde{x}}_{\mathrm{best}}^{\left(t\right)}+0.35u_{2}{\tilde{x}}_{i}^{\left(t\right)}+0.01\left(1-\rho_{t}\right)u_{3},$$

where $u_{1},u_{2}∼U\left(0,1\right)$, and $u_{3}∼U{\left(0,1\right)}^{D}$. Exactly one of the two candidates is evaluated for each target. The branch probability and the constants in (20) are distinct from α and remain fixed in the directional-weight sensitivity analysis.

The auxiliary stage is active in every complete sweep while evaluations remain. There is no comparison with a moving average of best-so-far fitness and no stagnation decision. After repair, a candidate replaces its target only if its objective value is strictly smaller. The resulting position is $x_{i}^{\left(t+1\right)}$; individuals outside the target set retain their post-parent positions.

### 3.3. Constraint Handling, Execution and Cost

Box-constrained candidates are clipped coordinate-wise to their bounds. For portfolio weights, negative coordinates are truncated and the nonnegative vector is normalized:

(21) $$u_{j}=\max\left(0,y_{j}\right),\;w_{j}=\left\{\begin{array}{cc} u_{j}/\sum_{k=1}^{D}u_{k}, & \sum_{k=1}^{D}u_{k}>0,\\ 1/D, & \mathrm{otherwise}. \end{array}\right.$$

This mapping guarantees nonnegative weights summing to one. The same repair is applied to all portfolio methods before an objective call.

**Figure 2:**
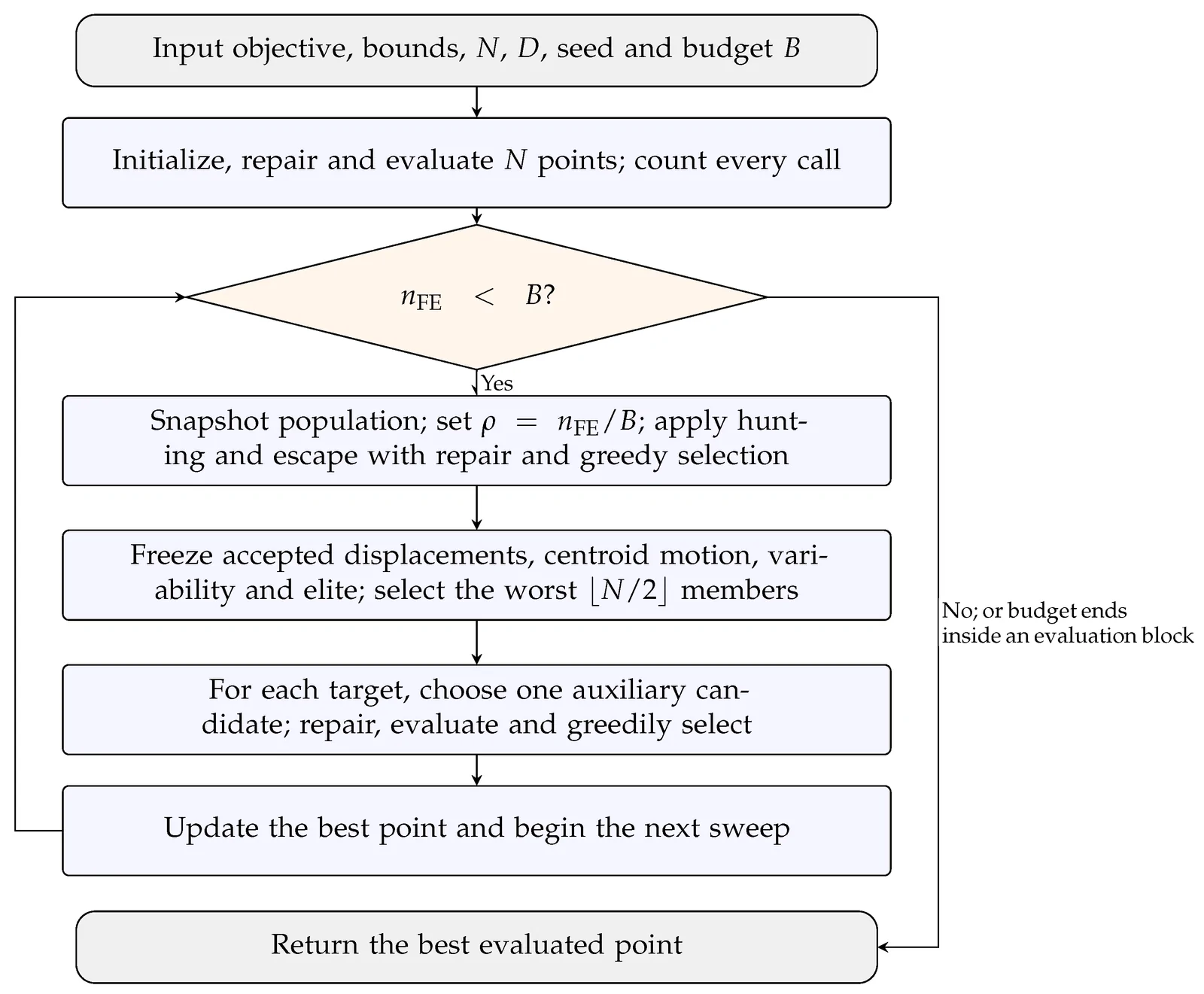
GT-SBOA execution flow. The auxiliary stage has no activation decision. All evaluation blocks enforce the same budget before each objective call.

Algorithm 2 and Figure 2 specify the sequence. Every evaluation passes through a single budget counter. Initialization uses *N* calls. Each complete sweep then uses 2N parent calls and *m* auxiliary calls, irrespective of how many trials are accepted. If the budget ends during either stage, the method returns the best evaluated solution immediately.
**Algorithm 2** GT-SBOA with explicit evaluation accounting1:**Input:** *f*, bounds, dimension *D*, population *N*, budget B≥N, seed2:Initialize by (13) and (14); apply portfolio repair when required3:Repair and evaluate *N* individuals; set $n_{\mathrm{FE}}=N$ and record the best point4:**while** $n_{\mathrm{FE}}<B$ **do**5:    Snapshot the population and set $\rho =n_{\mathrm{FE}}/B$6:    Apply hunting to all individuals, then escape to all individuals, using ρ for phase progress7:    Before each call: return the best point if $n_{\mathrm{FE}}=B$; otherwise repair, evaluate, increment and greedily select8:    Compute accepted displacements, centroid motion and displacement standard deviations9:    Freeze these quantities and the post-parent elite; select the worst ⌊N/2⌋ individuals10:    **for** each selected individual in stable index order **do**11:        Choose (19) or (20) with probabilities 0.65 and 0.3512:        Return the best point if $n_{\mathrm{FE}}=B$; otherwise repair, evaluate once, increment and greedily select13:    **end for**14:    Update the best evaluated point and start the next sweep15:**end while**16:**Output:** best evaluated point and objective value

Ignoring an incomplete final sweep, the number of completed sweeps is

(22) $$T_{\mathrm{GT}}=\left\lfloor \frac{B-N}{2N+\lfloor N/2\rfloor }\right\rfloor ,\;T_{\mathrm{SBOA}}=\left\lfloor \frac{B-N}{2N}\right\rfloor .$$

For N=30, GT-SBOA uses 75 calls per full sweep, compared with 60 for SBOA. At *B* = 300,000, this gives 3999 and 4999 complete sweeps, respectively; at B=5000, it gives 66 and 82. Thus, the auxiliary stage increases calls per sweep by 25% and reduces the number of complete sweeps by approximately 20% at large budgets. The comparison fixes evaluations, not iteration count.

Let $C_{f}$ be the objective-evaluation cost and $C_{\mathrm{prime}}\left(D\right)$ the one-time prime-search cost. Initialization costs $O(C_{\mathrm{prime}}+ND)$, excluding its objective calls. Parent updates, displacement calculation, candidate generation and repair cost O(ND) per sweep, and sorting the target set costs O(NlogN). The total cost is

(23) $$O\;\left(C_{\mathrm{prime}}+ND+T_{\mathrm{GT}}\left(ND+N\log N\right)+BC_{f}\right),$$

with O(ND) auxiliary storage. Linear-time selection can replace sorting when only the target set is needed.

## 4. Numerical Evaluation on CEC2017

### 4.1. Benchmark and Experimental Settings

The benchmark contains 29 CEC2017 functions—F1 and F3, F4–F10, F11–F20, and F21–F30—representing unimodal, multimodal, hybrid and composition landscapes [15]. F2 is excluded following the organizer’s implementation guidance [16]. Each function uses bounds ${\left[-100,100\right]}^{D}$, 30 independent runs and budget *B* = 10,000D. The error is the final objective value minus the known optimum; errors below $10^{-8}$ are set to zero before aggregation and testing.

The D=30 comparison includes GT-SBOA, SBOA, UTFSBOA, ISBOA, RIME, ECO, PO, SBO, DE, PSO and GWO. The higher-dimensional comparison at D=50 and 100 retains the four SBOA-family methods to examine scaling within that family. Population size is N=30; for the two-population SBO formulation, this means 15 two-member families. Initial candidate evaluations, opposition candidates, unsuccessful trials and repaired trials all consume the stated budget. The original box initializers of the comparison methods are retained, including the 2N selection pool of ISBOA; its entire pool is charged to the budget. Each algorithm has an independent random stream.

Table 2 states the coefficients and schedules. Progress-based schedules use consumed evaluations divided by *B*, so added operators do not implicitly receive extra iterations. ISBOA’s stagnation allowance is expressed as an evaluation fraction, with a minimum of five complete sweeps; all calls made by its escape step remain within *B*. Implementations cache objective values for unchanged candidates and stop before any call exceeding the budget. The timing protocol measures the algorithmic initialization and search updates under standardized single-thread execution, excluding file I/O and compilation, and uses an untimed warm-up with randomized run order. The algorithmic runtime summaries across dimensions are reported alongside the performance evaluation in Section 4.2.

The timing calibration environment uses a workstation with an Intel Core Ultra 7 265K processor (20 cores; maximum frequency 5.6 GHz), 30.75 GiB of system-visible RAM, and Ubuntu 24.04.3 LTS. MATLAB R2025b Update 4 (25.2.0.3150157) uses Intel oneAPI MKL 2024.1 with LAPACK 3.11.0. Each calibration run uses one computational thread and no parallel pool or GPU; the same environment and timing boundaries are specified for the complete comparison.

### 4.2. Performance and Statistical Comparison

For each function, mean error and sample standard deviation are calculated from the 30 final values. Mean ranks are computed within each function and then averaged; equal means receive midranks. A best count includes every tied minimum. Mean W/T/L counts compare mean errors directly and are distinct from significant W/T/L counts.

Independent run distributions are compared using two-sided Wilcoxon rank-sum tests with tie correction and continuity correction. For each GT-SBOA–comparator pair at a given dimension, Holm adjustment controls the family of 29 function-level tests at α=0.05. The direction of a significant difference is expressed by

(24) $$A_{\mathrm{lower}}=\mathrm{Pr}\left(E_{\mathrm{GT}}<E_{A}\right)+\frac{1}{2}\mathrm{Pr}\left(E_{\mathrm{GT}}=E_{A}\right),$$

estimated from all between-sample pairs. A significant win requires adjusted p<0.05 and $A_{\mathrm{lower}}>0.5$; a loss requires adjusted p<0.05 and $A_{\mathrm{lower}}<0.5$. Otherwise, the comparison is recorded as a tie, meaning that no difference is detected by this procedure.

Table 3 gives the complete D=30 results. GT-SBOA has the lowest average rank (2.4138) and the lowest mean error on 14 functions. Against SBOA, its mean W/T/L is 16/0/13, and its significant W/T/L is 16/4/9. Reporting both counts distinguishes numerical ordering from evidence of a distributional difference. Table 4 presents the corresponding Holm-adjusted, two-sided Wilcoxon rank-sum *p*-values and pairwise test decisions across all 29 functions.

Across-function analysis treats the functions as blocks and ranks the algorithm means within each block [18]. The Friedman statistic at D=30 is $\chi_{F}^{2}=170.1818$ with 10 degrees of freedom. Control-versus-comparator rank differences are standardized by $\sqrt{k(k+1)/(6n)}$, where n=29, and *k* is the number of algorithms at that dimension. Two-sided normal-approximation probabilities are Holm-adjusted over the k−1 control comparisons, separately from the run-level families. Table 5 gives these aggregate comparisons.

### 4.3. Component Contributions and Directional Weight

The ablation is a two-factor comparison: SBOA uses neither component; GPS-SBOA uses the corrected initializer only; T-SBOA uses the complete auxiliary update with uniform initialization; GT-SBOA uses both. All variants use the same population, bounds, objective definition, repair, evaluation limit and 30-run protocol. The GT-SBOA and SBOA records are exactly those used in the main D=30 comparison. Their mean and significant W/T/L therefore agree between Table 3 and Table 6. Average ranks can change because they are calculated within different comparator sets.

Table 6 places the complete configuration first by mean rank (2.0345). Its comparison with each single-component variant describes the effect of removing that component under an equal evaluation budget. The trend-only variant includes the primary and alternative candidate branches, targeting rule and decaying perturbations as one defined component.

The directional-weight comparison uses α∈{0,0.35,0.50,0.65,0.80,1} with all other coefficients fixed. The endpoints isolate centroid and individual displacement, respectively. The default α=0.65 uses the same records as the main experiment. Table 7 reports ranks within the six settings; the mixed setting α=0.65 has the lowest mean rank, with α=0.50 close behind. This comparison describes the sensitivity of the parameter α.

### 4.4. Dimension Dependence and Computational Cost

Table 8 summarizes the dimension-specific comparisons at D=50 and D=100. The four-method mean rank of GT-SBOA is 1.9483 at D=50 and 2.5690 at D=100. At D=100, ISBOA ranks first, UTFSBOA second, GT-SBOA third and SBOA fourth.

Table 9 reports $A_{\mathrm{lower}}$ and pointwise bootstrap intervals for F3 at all three dimensions. The full effect estimates and adjusted probabilities are retained with the function-level records. Table 10 reports wall-clock summaries separately from the evaluation accounting in (22). The auxiliary stage spends a fixed share of evaluations on displacement-guided candidates; its utility depends on whether those candidates compensate for the parent sweeps that are displaced.

## 5. Portfolio Optimization Case Study

### 5.1. Model and Common Experimental Protocol

The case study uses the long-only, fully invested mean–variance model [19,20]. For asset weights w, expected returns μ and covariance matrix Σ,

(25) $$R\left(w\right)=\mu^{⊤}w,\;V\left(w\right)=w^{⊤}\Sigma w,$$

subject to $\sum_{j=1}^{D}w_{j}=1$ and $w_{j}\geq 0$. Risk in frontier-distance metrics is the standard deviation $\sigma \left(w\right)=\sqrt{V(w)}$; the objective retains variance. The five OR-Library instances [21] contain 31 (Hang Seng), 85 (DAX 100), 89 (FTSE 100), 98 (S&P 100) and 225 (Nikkei 225) assets. Covariances are reconstructed from the supplied standard deviations and correlations. The reference-frontier files contain mean return and variance, so their second coordinate is square-rooted before risk-standard-deviation distances are calculated.

The scalarized mean–variance objective is

(26) $$f_{\lambda }\left(w\right)=\lambda V\left(w\right)-\left(1-\lambda \right)R\left(w\right),\;\lambda \in \left\{0,0.05,\ldots ,1\right\}.$$

The same objective and scalarization grid are used for every algorithm. Reference portfolios are not supplied as initial solutions.

The main comparison evaluates all eleven complete algorithms in Table 2. GT-SBOA retains its GPS initializer: generate the N=30 points in ${\left[0,1\right]}^{D}$ using (13), then apply (21). Methods with ordinary random initialization draw points uniformly in ${\left[0,1\right]}^{D}$ before the same repair. Methods with a published specialized initializer retain it under the common feasible encoding and budget, including the charged 2N opposition-selection pool of ISBOA. SBO allocates its 30 members to 15 families. No method receives externally added basis portfolios or an application-specific candidate pool. GT-SBOA initializes directly from its 30 GPS points without fitness-based preliminary selection.

The common rules concern the objective, feasible encoding, repair, population size, scalarization grid and total evaluation budget. Initialization remains an algorithm component in the main comparison. Table 11 compares the proposed search updates with the parent SBOA under the same random initialization rule, separating search performance from the GPS starting population. All stochastic draws use independent algorithm/run streams.

Each scalarization has a budget of 5000 evaluations, including every initialization and selection-pool evaluation. Thirty independent complete frontiers are assembled per algorithm and dataset, each from one run at every one of the 21 weights. A complete frontier, rather than an individual scalarization, is the replication unit for statistical analysis. The simplex repair in (21) is identical across methods. The within-sample convex model provides a transparent benchmark for frontier approximation.

### 5.2. Frontier Accuracy, Coverage and Risk Interpretation

Let *P* be the supplied reference frontier in (σ,R) coordinates, and let *Q* be the nondominated points of one complete run. With $\Delta_{R}=R_{\max}-R_{\min}$, the common minimization-coordinate transformation is

(27) $$z\left(\sigma ,R\right)=\left(\frac{\sigma -\sigma_{\min}}{\Delta_{\sigma }},\frac{R_{\max}-R}{\Delta_{R}}\right),\;\Delta_{\sigma }=\sigma_{\max}-\sigma_{\min}.$$

Distances are Euclidean in these normalized coordinates. The root-mean-square generational distance and inverted generational distance are

(28) $$\mathrm{GD}\left(Q,P\right)={\left[\frac{1}{\left|Q\right|}\sum_{q\in Q}\min_{p\in P}{∥z\left(q\right)-z\left(p\right)∥}_{2}^{2}\right]}^{1/2},$$

(29) $$\mathrm{IGD}\left(Q,P\right)={\left[\frac{1}{\left|P\right|}\sum_{p\in P}\min_{q\in Q}{∥z\left(p\right)-z\left(q\right)∥}_{2}^{2}\right]}^{1/2}.$$

GD measures proximity of returned points to the reference, while IGD also penalizes reference regions without nearby returned points. Lower values are preferred for both metrics. Every method uses the same reference frontier and normalization.

Table 12 and Table 13 report mean GD and IGD with sample standard deviations over complete frontiers. GT-SBOA has average ranks of 1.40 and 1.00, respectively.

To express approximation error in financial units, the reference risk is interpolated at the expected return of each candidate portfolio. The mean excess risk-standard-deviation gap is

(30) $$\Delta_{\sigma }^{\mathrm{bp}}=\frac{10^{4}}{21}\sum_{k=1}^{21}\left[\sigma \left(w_{k}\right)-\sigma_{P}\left(R\left(w_{k}\right)\right)\right],$$

where $\sigma_{P}\left(R\right)$ is the piecewise-linear interpolation of the reference frontier. The calculation is restricted to candidate returns within the reference return range; if any fall outside it, the average uses the number of valid points instead of 21 and that count is recorded. Table 14 gives this quantity for the four SBOA-family methods. For example, the port1 gaps for GT-SBOA and SBOA are 0.043 and 0.049 basis points, respectively; the difference is 0.006 basis points in risk standard deviation at matched expected return.

The application results compare allocation quality within a fixed in-sample model and evaluation budget. Table 15 reports significant wins, ties and losses against each comparator. The independent-sample tests use one Holm family covering 100 comparisons across five datasets, ten comparators and two metrics.

To check the role of the starting population in the comparison with the parent algorithm, Table 11 uses the same random initialization rule for GT-SBOA’s search updates and SBOA. Both draw independent uniform points in ${\left[0,1\right]}^{D}$ and apply (21), without preliminary selection. The GT-SBOA (RI) label identifies the proposed updates with GPS disabled. Population size, scalarizations and evaluation budget remain unchanged; the SBOA records are reused from the main comparison. The control has a separate Holm family of ten comparisons across five datasets and two metrics. Its significant W/T/L against SBOA is 4/0/1 for GD and 5/0/0 for IGD. The main protocol evaluates complete algorithms, while the parent-algorithm control checks search performance under a common initialization rule.

## 6. Conclusions

GT-SBOA combines a dimension-admissible good-point initializer with an auxiliary direction derived from accepted individual and centroid displacements. The corrected generator removes repeated cosine coordinates. The auxiliary stage is explicitly active in every complete sweep, and its objective calls share the same budget as the hunting and escape updates. This formulation makes the allocation of evaluations and the resulting reduction in parent sweeps explicit.

The CEC2017 comparison places GT-SBOA first by mean rank at D=30 and D=50 within their respective comparator sets, while the D=100 comparison places it third behind ISBOA and UTFSBOA. At D=30, its significant W/T/L against SBOA is 16/4/9. The component comparison uses the same parent and complete-method records as the main benchmark, linking its conclusions to a consistent experimental basis. The main portfolio comparison retains GPS as part of GT-SBOA and removes the application-specific candidate pool and externally added basis portfolios. A common-initialization control against SBOA isolates the proposed search updates, while matched-return risk gaps provide a financial interpretation beyond normalized distance.

The resulting evidence supports evaluating displacement reuse as an allocation of limited search effort whose value depends on dimension and landscape. Future development can examine selective targeting and adaptive directional weights, as well as portfolio extensions with additional operational constraints.

## Figures and Tables

**Table 1:** Direct verification of the corrected initialization for N=30. A duplicate pair means that all 30 normalized coordinates agree within $10^{-10}$.

*D*	Prime *p*	Distinct $r_{j}$	Duplicate Pairs
30	67	30	0
50	103	50	0
100	211	100	0

**Table 2:** Algorithm settings for the revised comparison. Common budgets and population conventions are specified in Section 4.1. ρ denotes consumed-budget progress. Published internal schedules are retained unless an adaptation is stated.

Algorithm	Settings
GT-SBOA	α=0.65, target fraction 0.5, $p_{\mathrm{alt}}=0.35$; coefficients in (19)–(20); corrected GPS for box problems.
SBOA	Gaussian Mantegna sampling, Lévy exponent 1.5, multiplier 0.5; escape branch probability 0.5; uniform $R_{2}$.
UTFSBOA	Cauchy and Gaussian scales 1; crossover probability 0.5; directional sign in {−1,1}; density and energy schedules from [12]. Mutation-mixture probability 0.5 is the fixed implementation choice.
ISBOA	Logistic coefficient 4, Tent breakpoint 0.5; F=0.5+0.3U(0,1), CR=0.8−0.5ρ, Lévy exponent 1.5; nonlinear factor ${\left(1-\rho^{2}\right)}^{2}U\left(0,1\right)$; stagnation allowance 0.1B evaluations, with a minimum of five complete sweeps.
RIME	Soft-rime parameter W=5; exploration probability $\sqrt{\rho }$; hard-rime probability follows normalized fitness [6].
ECO	H=0.5, $G_{1}=0.2$, $G_{2}=0.1$; logistic initialization coefficient 4; stage order and schedules from the authors’ implementation [7].
PO	Four behavior choices with probability 1/4 each; Lévy exponent 1.5; communication branch probability 0.5, step weight U(0,0.2), angle U(0,π) [8].
SBO	Two members per family: 15 families and 30 initial members; Gaussian interaction weights; adaptive $w_{3}$ and $w_{4}∼U\left(-w_{3},w_{3}\right)$ as in [9].
DE	DE/rand/1/bin; F=0.5, CR=0.9; at least one forced mutant coordinate.
PSO	Global-best topology; inertia 0.9−0.5ρ, $c_{1}=c_{2}=1.5$; initial velocity range ±0.1(ub−lb) and clamp ±0.2(ub−lb).
GWO	Three leaders; a=2(1−ρ) and independent uniform coefficient vectors [17].

**Table 3:** Results of GT-SBOA and the comparison algorithms on the CEC2017 benchmark functions at D=30. Mean (Avg) and sample standard deviation (Std) over 30 independent records; errors below $10^{-8}$ are treated as zero. Bold indicates the lowest mean.

	**F1**		**F3**		**F4**	
** Algo.**	**Avg**	**Std**	**Avg**	**Std**	**Avg**	**Std**
GT-SBOA	0	0	$1.8001\times 10^{1}$	$9.6230\times 10^{0}$	$6.8519\times 10^{1}$	$2.5282\times 10^{1}$
SBOA	$2.5289\times 10^{3}$	$2.1360\times 10^{3}$	$3.1926\times 10^{1}$	$5.3267\times 10^{-1}$	$5.1552\times 10^{1}$	$1.8815\times 10^{1}$
UTFSBOA	$4.8682\times 10^{1}$	$8.9609\times 10^{1}$	$3.6652\times 10^{1}$	$1.0728\times 10^{1}$	$1.4350\times 10^{2}$	$2.8782\times 10^{1}$
ISBOA	$3.4853\times 10^{0}$	$9.2840\times 10^{0}$	$2.7660\times 10^{1}$	$9.5784\times 10^{0}$	$6.1186\times 10^{1}$	$1.9326\times 10^{1}$
RIME	$1.9578\times 10^{4}$	$1.0681\times 10^{4}$	$5.8510\times 10^{1}$	$2.6277\times 10^{1}$	$1.5142\times 10^{2}$	$3.0279\times 10^{1}$
ECO	$6.4443\times 10^{3}$	$5.5232\times 10^{3}$	$4.9452\times 10^{1}$	$2.5728\times 10^{1}$	$7.0954\times 10^{2}$	$2.3281\times 10^{2}$
PO	$2.6825\times 10^{3}$	$2.1095\times 10^{3}$	$3.8650\times 10^{1}$	$1.6787\times 10^{1}$	$1.9936\times 10^{2}$	$7.9786\times 10^{1}$
SBO	$8.8840\times 10^{-2}$	$1.5548\times 10^{-1}$	$4.4673\times 10^{1}$	$2.1994\times 10^{1}$	$2.1479\times 10^{2}$	$5.3105\times 10^{1}$
DE	$6.3954\times 10^{7}$	$8.2140\times 10^{7}$	$4.3600\times 10^{1}$	$2.1464\times 10^{1}$	$1.0198\times 10^{2}$	$8.2051\times 10^{1}$
PSO	$1.6330\times 10^{9}$	$1.0733\times 10^{9}$	$2.5062\times 10^{2}$	$2.1669\times 10^{2}$	$4.2280\times 10^{3}$	$2.6362\times 10^{3}$
GWO	$8.8053\times 10^{9}$	$9.7708\times 10^{8}$	$5.6321\times 10^{2}$	$4.8931\times 10^{1}$	$7.2645\times 10^{3}$	$5.7032\times 10^{2}$
	**F5**		**F6**		**F7**	
**Algo.**	**Avg**	**Std**	**Avg**	**Std**	**Avg**	**Std**
GT-SBOA	$2.2882\times 10^{-6}$	$2.2532\times 10^{-6}$	$9.3054\times 10^{1}$	$2.2669\times 10^{1}$	$7.3107\times 10^{1}$	$1.5181\times 10^{1}$
SBOA	$1.9458\times 10^{-5}$	$1.9591\times 10^{-5}$	$9.2385\times 10^{1}$	$1.2550\times 10^{1}$	$1.4613\times 10^{2}$	$4.1726\times 10^{1}$
UTFSBOA	$4.7447\times 10^{-5}$	$5.4797\times 10^{-5}$	$2.0662\times 10^{2}$	$5.3234\times 10^{1}$	$3.6099\times 10^{2}$	$7.8503\times 10^{1}$
ISBOA	$6.7200\times 10^{-5}$	$6.6098\times 10^{-5}$	$1.3490\times 10^{2}$	$3.3064\times 10^{1}$	$1.7375\times 10^{2}$	$5.7914\times 10^{1}$
RIME	$2.6007\times 10^{-3}$	$1.6900\times 10^{-3}$	$2.2385\times 10^{2}$	$3.8218\times 10^{1}$	$2.3657\times 10^{2}$	$4.5170\times 10^{1}$
ECO	$4.4788\times 10^{-4}$	$5.2564\times 10^{-4}$	$7.5698\times 10^{2}$	$2.3405\times 10^{2}$	$3.4353\times 10^{2}$	$5.4552\times 10^{1}$
PO	$1.0360\times 10^{-4}$	$6.1971\times 10^{-5}$	$2.6450\times 10^{2}$	$1.4468\times 10^{2}$	$1.4920\times 10^{2}$	$2.5896\times 10^{1}$
SBO	$9.5332\times 10^{-5}$	$1.1699\times 10^{-4}$	$2.6092\times 10^{2}$	$7.1856\times 10^{1}$	$2.4699\times 10^{2}$	$4.9596\times 10^{1}$
DE	$4.1623\times 10^{-7}$	$6.9584\times 10^{-7}$	$1.7315\times 10^{2}$	$1.8992\times 10^{2}$	$9.4731\times 10^{1}$	$1.9526\times 10^{1}$
PSO	$1.0279\times 10^{-4}$	$3.3624\times 10^{-4}$	$2.4441\times 10^{5}$	$1.8115\times 10^{5}$	$1.7679\times 10^{2}$	$4.7760\times 10^{1}$
GWO	$6.0417\times 10^{-3}$	$1.8603\times 10^{-3}$	$3.0164\times 10^{5}$	$2.5671\times 10^{4}$	$3.2651\times 10^{2}$	$2.4712\times 10^{1}$
	**F8**		**F9**		**F10**	
**Algo.**	**Avg**	**Std**	**Avg**	**Std**	**Avg**	**Std**
GT-SBOA	$5.9687\times 10^{-1}$	$3.6534\times 10^{-1}$	$1.6595\times 10^{3}$	$2.9689\times 10^{2}$	$4.2947\times 10^{2}$	$4.7110\times 10^{2}$
SBOA	0	0	$3.3160\times 10^{3}$	$7.5533\times 10^{2}$	$2.6584\times 10^{4}$	$9.6183\times 10^{3}$
UTFSBOA	$2.3451\times 10^{-4}$	$8.3677\times 10^{-5}$	$3.5043\times 10^{3}$	$8.6977\times 10^{2}$	$2.0081\times 10^{3}$	$1.2218\times 10^{3}$
ISBOA	$1.1354\times 10^{-9}$	$3.4770\times 10^{-9}$	$3.9569\times 10^{3}$	$8.7226\times 10^{2}$	$5.3647\times 10^{3}$	$2.4787\times 10^{3}$
RIME	$8.2898\times 10^{0}$	$4.4244\times 10^{0}$	$3.5278\times 10^{3}$	$6.9864\times 10^{2}$	$3.0757\times 10^{4}$	$2.1098\times 10^{4}$
ECO	$8.7007\times 10^{0}$	$2.8251\times 10^{0}$	$4.0673\times 10^{3}$	$1.0224\times 10^{3}$	$3.4481\times 10^{3}$	$3.9416\times 10^{3}$
PO	$1.9151\times 10^{0}$	$1.0181\times 10^{0}$	$3.6213\times 10^{3}$	$3.3687\times 10^{2}$	$3.6134\times 10^{3}$	$3.3507\times 10^{3}$
SBO	$5.3399\times 10^{0}$	$1.6248\times 10^{0}$	$3.0114\times 10^{3}$	$5.8242\times 10^{2}$	$9.7661\times 10^{2}$	$4.5588\times 10^{2}$
DE	$1.1473\times 10^{0}$	$6.1669\times 10^{-1}$	$2.9194\times 10^{3}$	$8.6617\times 10^{2}$	$5.9620\times 10^{3}$	$5.0681\times 10^{3}$
PSO	$3.3866\times 10^{0}$	$2.1665\times 10^{0}$	$2.8245\times 10^{3}$	$4.6100\times 10^{2}$	$9.9660\times 10^{4}$	$7.0764\times 10^{4}$
GWO	$6.8043\times 10^{0}$	$1.0741\times 10^{0}$	$5.5577\times 10^{3}$	$4.1476\times 10^{2}$	$1.7338\times 10^{7}$	$8.6216\times 10^{6}$
**Algo.**	**Avg**	**Std**	**Avg**	**Std**	**Avg**	**Std**
GT-SBOA	$2.8183\times 10^{3}$	$2.4306\times 10^{3}$	$2.8887\times 10^{3}$	$3.2779\times 10^{3}$	$3.9032\times 10^{2}$	$5.2781\times 10^{2}$
SBOA	$1.9142\times 10^{5}$	$2.2265\times 10^{5}$	$9.3048\times 10^{3}$	$4.5652\times 10^{3}$	$7.7525\times 10^{4}$	$3.3144\times 10^{4}$
UTFSBOA	$3.4186\times 10^{4}$	$2.8324\times 10^{4}$	$9.8548\times 10^{3}$	$9.1414\times 10^{3}$	$3.3568\times 10^{4}$	$2.0773\times 10^{4}$
ISBOA	$8.1998\times 10^{3}$	$7.8039\times 10^{3}$	$5.3035\times 10^{3}$	$3.2788\times 10^{3}$	$1.9433\times 10^{4}$	$1.5504\times 10^{4}$
RIME	$2.5392\times 10^{5}$	$3.3861\times 10^{5}$	$1.9093\times 10^{5}$	$4.7493\times 10^{5}$	$3.2106\times 10^{6}$	$3.8939\times 10^{6}$
ECO	$9.4631\times 10^{3}$	$6.9436\times 10^{3}$	$9.1469\times 10^{3}$	$9.4671\times 10^{3}$	$1.9140\times 10^{4}$	$1.8064\times 10^{4}$
PO	$1.7051\times 10^{4}$	$1.2250\times 10^{4}$	$6.1592\times 10^{4}$	$2.0605\times 10^{5}$	$1.3389\times 10^{5}$	$6.2997\times 10^{4}$
SBO	$5.0599\times 10^{3}$	$4.5164\times 10^{3}$	$4.4896\times 10^{3}$	$4.2714\times 10^{3}$	$6.9362\times 10^{3}$	$5.0868\times 10^{3}$
DE	$1.3966\times 10^{4}$	$9.6987\times 10^{3}$	$5.7615\times 10^{3}$	$4.1915\times 10^{3}$	$1.5903\times 10^{4}$	$1.6638\times 10^{4}$
PSO	$7.0966\times 10^{8}$	$1.5321\times 10^{9}$	$5.6248\times 10^{7}$	$9.4513\times 10^{7}$	$3.3797\times 10^{4}$	$2.1824\times 10^{4}$
GWO	$4.8678\times 10^{8}$	$1.8846\times 10^{8}$	$4.5146\times 10^{8}$	$1.3951\times 10^{8}$	$1.6370\times 10^{6}$	$1.1290\times 10^{6}$
	**F14**		**F15**		**F16**	
**Algo.**	**Avg**	**Std**	**Avg**	**Std**	**Avg**	**Std**
GT-SBOA	$1.6021\times 10^{3}$	$1.3097\times 10^{3}$	$3.3276\times 10^{2}$	$1.8949\times 10^{2}$	$2.8992\times 10^{2}$	$2.7570\times 10^{2}$
SBOA	$5.5548\times 10^{4}$	$4.0566\times 10^{4}$	$1.2598\times 10^{1}$	$1.2943\times 10^{1}$	$1.4376\times 10^{4}$	$6.5690\times 10^{3}$
UTFSBOA	$1.2585\times 10^{4}$	$8.4572\times 10^{3}$	$3.6034\times 10^{1}$	$3.6051\times 10^{1}$	$1.0410\times 10^{3}$	$6.2448\times 10^{2}$
ISBOA	$1.3665\times 10^{4}$	$9.5096\times 10^{3}$	$2.5803\times 10^{1}$	$2.1841\times 10^{1}$	$1.1191\times 10^{3}$	$9.6916\times 10^{2}$
RIME	$5.0338\times 10^{4}$	$4.6178\times 10^{4}$	$1.0129\times 10^{4}$	$1.1383\times 10^{4}$	$1.2151\times 10^{3}$	$3.7836\times 10^{2}$
ECO	$1.6679\times 10^{4}$	$9.1680\times 10^{3}$	$4.6002\times 10^{2}$	$3.7349\times 10^{2}$	$1.3209\times 10^{3}$	$6.1400\times 10^{2}$
PO	$9.3421\times 10^{3}$	$6.1481\times 10^{3}$	$7.1109\times 10^{2}$	$6.4272\times 10^{2}$	$2.5607\times 10^{3}$	$4.2346\times 10^{3}$
SBO	$5.3193\times 10^{3}$	$3.6870\times 10^{3}$	$2.4356\times 10^{2}$	$1.8572\times 10^{2}$	$5.9581\times 10^{2}$	$4.7779\times 10^{2}$
DE	$8.6824\times 10^{3}$	$4.0895\times 10^{3}$	$3.6417\times 10^{4}$	$5.9954\times 10^{4}$	$5.2607\times 10^{2}$	$1.1745\times 10^{3}$
PSO	$3.0093\times 10^{6}$	$4.8396\times 10^{6}$	$5.9685\times 10^{5}$	$6.7927\times 10^{5}$	$9.3290\times 10^{4}$	$1.0150\times 10^{5}$
GWO	$9.5429\times 10^{7}$	$4.4583\times 10^{7}$	$6.7075\times 10^{6}$	$3.6986\times 10^{6}$	$3.0434\times 10^{6}$	$9.2451\times 10^{6}$
	**F17**		**F18**		**F19**	
**Algo.**	**Avg**	**Std**	**Avg**	**Std**	**Avg**	**Std**
GT-SBOA	$4.2145\times 10^{2}$	$4.0230\times 10^{2}$	$2.0381\times 10^{1}$	$7.7327\times 10^{0}$	$1.9344\times 10^{2}$	$2.0406\times 10^{2}$
SBOA	$1.0613\times 10^{5}$	$6.5347\times 10^{4}$	$1.8418\times 10^{4}$	$1.4438\times 10^{4}$	$9.4029\times 10^{1}$	$5.8197\times 10^{1}$
UTFSBOA	$6.2258\times 10^{3}$	$7.1000\times 10^{3}$	$1.4461\times 10^{4}$	$1.1938\times 10^{4}$	$1.2387\times 10^{2}$	$9.1634\times 10^{1}$
ISBOA	$1.6163\times 10^{4}$	$1.4535\times 10^{4}$	$1.7803\times 10^{4}$	$1.5818\times 10^{4}$	$2.2297\times 10^{2}$	$1.6894\times 10^{2}$
RIME	$2.5404\times 10^{5}$	$4.4477\times 10^{5}$	$3.8519\times 10^{4}$	$6.3164\times 10^{4}$	$2.7187\times 10^{2}$	$1.4616\times 10^{2}$
ECO	$2.1139\times 10^{4}$	$1.9860\times 10^{4}$	$1.7415\times 10^{4}$	$1.9589\times 10^{4}$	$5.3890\times 10^{2}$	$2.5237\times 10^{2}$
PO	$1.2562\times 10^{5}$	$4.4610\times 10^{4}$	$1.1391\times 10^{4}$	$9.2190\times 10^{3}$	$1.9741\times 10^{2}$	$1.4496\times 10^{2}$
SBO	$3.7552\times 10^{3}$	$6.2725\times 10^{3}$	$1.1530\times 10^{4}$	$9.8725\times 10^{3}$	$3.0118\times 10^{2}$	$1.7192\times 10^{2}$
DE	$1.0097\times 10^{4}$	$9.1522\times 10^{3}$	$3.2025\times 10^{5}$	$3.9003\times 10^{5}$	$1.6113\times 10^{2}$	$1.1501\times 10^{2}$
PSO	$9.3296\times 10^{4}$	$1.4906\times 10^{5}$	$2.9300\times 10^{9}$	$3.6274\times 10^{9}$	$2.8065\times 10^{2}$	$1.6034\times 10^{2}$
GWO	$1.3319\times 10^{6}$	$4.1543\times 10^{5}$	$9.4497\times 10^{9}$	$3.1507\times 10^{9}$	$1.9279\times 10^{3}$	$3.2032\times 10^{2}$
	**F20**		**F21**		**F22**	
**Algo.**	**Avg**	**Std**	**Avg**	**Std**	**Avg**	**Std**
GT-SBOA	$2.1170\times 10^{2}$	$6.6603\times 10^{1}$	$1.7984\times 10^{2}$	$9.5929\times 10^{0}$	$2.3745\times 10^{2}$	$9.5077\times 10^{1}$
SBOA	$1.0595\times 10^{2}$	$5.0715\times 10^{1}$	$1.6240\times 10^{2}$	$3.4906\times 10^{0}$	$2.0160\times 10^{2}$	$3.5542\times 10^{1}$
UTFSBOA	$1.2227\times 10^{2}$	$5.1623\times 10^{1}$	$1.0672\times 10^{2}$	$1.3145\times 10^{0}$	$2.2701\times 10^{2}$	$1.0766\times 10^{2}$
ISBOA	$1.8488\times 10^{2}$	$8.7595\times 10^{1}$	$8.9366\times 10^{1}$	$1.2455\times 10^{0}$	$4.4573\times 10^{2}$	$1.5962\times 10^{2}$
RIME	$4.2591\times 10^{2}$	$4.4519\times 10^{2}$	$1.6033\times 10^{2}$	$2.1685\times 10^{0}$	$5.7286\times 10^{2}$	$7.9251\times 10^{2}$
ECO	$6.3944\times 10^{2}$	$3.2611\times 10^{2}$	$2.0920\times 10^{2}$	$1.8330\times 10^{1}$	$3.0771\times 10^{2}$	$3.7341\times 10^{2}$
PO	$3.1047\times 10^{2}$	$9.9511\times 10^{1}$	$1.9434\times 10^{2}$	$1.2478\times 10^{1}$	$2.5906\times 10^{2}$	$1.1232\times 10^{2}$
SBO	$3.0537\times 10^{2}$	$2.4640\times 10^{2}$	$1.6078\times 10^{2}$	$1.6476\times 10^{0}$	$2.6876\times 10^{2}$	$1.2976\times 10^{2}$
DE	$3.7410\times 10^{2}$	$2.6095\times 10^{2}$	$1.9601\times 10^{2}$	$1.8406\times 10^{1}$	$1.5582\times 10^{3}$	$2.9015\times 10^{3}$
PSO	$2.3651\times 10^{3}$	$1.3554\times 10^{3}$	$2.9476\times 10^{2}$	$7.9951\times 10^{1}$	$1.0997\times 10^{4}$	$8.1536\times 10^{3}$
GWO	$6.2049\times 10^{3}$	$9.4264\times 10^{2}$	$2.8349\times 10^{2}$	$1.3099\times 10^{1}$	$1.1787\times 10^{4}$	$1.7285\times 10^{3}$
**Algo.**	**Avg**	**Std**	**Avg**	**Std**	**Avg**	**Std**
GT-SBOA	$2.2552\times 10^{2}$	$5.1088\times 10^{1}$	$4.2186\times 10^{2}$	$1.9171\times 10^{0}$	$8.2853\times 10^{2}$	$1.8227\times 10^{0}$
SBOA	$2.0000\times 10^{2}$	$8.3589\times 10^{-12}$	$4.2071\times 10^{2}$	$1.5468\times 10^{-1}$	$8.2786\times 10^{2}$	$1.7799\times 10^{-2}$
UTFSBOA	$2.4362\times 10^{2}$	$9.0732\times 10^{1}$	$2.8123\times 10^{2}$	$3.2367\times 10^{0}$	$7.5917\times 10^{2}$	$4.5953\times 10^{0}$
ISBOA	$2.7562\times 10^{2}$	$8.9970\times 10^{1}$	$3.7809\times 10^{2}$	$4.8856\times 10^{0}$	$7.1594\times 10^{2}$	$3.6439\times 10^{0}$
RIME	$5.3371\times 10^{2}$	$7.6559\times 10^{2}$	$4.4795\times 10^{2}$	$2.9727\times 10^{1}$	$8.3265\times 10^{2}$	$1.9011\times 10^{1}$
ECO	$3.5721\times 10^{2}$	$3.0075\times 10^{2}$	$4.3652\times 10^{2}$	$1.1708\times 10^{1}$	$8.5807\times 10^{2}$	$2.0937\times 10^{1}$
PO	$2.2318\times 10^{2}$	$7.3168\times 10^{1}$	$4.3820\times 10^{2}$	$1.9612\times 10^{1}$	$8.4954\times 10^{2}$	$1.8050\times 10^{1}$
SBO	$3.2499\times 10^{2}$	$3.0458\times 10^{2}$	$4.2973\times 10^{2}$	$1.0436\times 10^{1}$	$8.3056\times 10^{2}$	$1.0319\times 10^{1}$
DE	$6.7226\times 10^{2}$	$7.0572\times 10^{2}$	$4.2575\times 10^{2}$	$5.4170\times 10^{0}$	$8.4271\times 10^{2}$	$1.7755\times 10^{1}$
PSO	$6.5882\times 10^{3}$	$2.3467\times 10^{3}$	$4.9695\times 10^{2}$	$5.9890\times 10^{1}$	$1.1513\times 10^{3}$	$2.6066\times 10^{2}$
GWO	$7.9224\times 10^{3}$	$5.5246\times 10^{2}$	$6.9069\times 10^{2}$	$5.4254\times 10^{1}$	$9.9722\times 10^{2}$	$2.3667\times 10^{1}$
	**F26**		**F27**		**F28**	
**Algo.**	**Avg**	**Std**	**Avg**	**Std**	**Avg**	**Std**
GT-SBOA	$5.1760\times 10^{2}$	$9.2742\times 10^{0}$	$8.5148\times 10^{1}$	$9.2766\times 10^{1}$	$3.8611\times 10^{3}$	$2.6008\times 10^{3}$
SBOA	$5.0620\times 10^{2}$	$3.8488\times 10^{0}$	$3.6698\times 10^{-1}$	$6.7873\times 10^{-1}$	$1.9790\times 10^{4}$	$8.1013\times 10^{3}$
UTFSBOA	$4.7226\times 10^{2}$	$1.3645\times 10^{1}$	$1.8713\times 10^{1}$	$3.3333\times 10^{1}$	$4.8784\times 10^{3}$	$1.7612\times 10^{3}$
ISBOA	$4.3452\times 10^{2}$	$1.2271\times 10^{1}$	$7.3295\times 10^{0}$	$9.3142\times 10^{0}$	$5.2007\times 10^{3}$	$1.6007\times 10^{3}$
RIME	$5.3663\times 10^{2}$	$2.1690\times 10^{1}$	$1.5948\times 10^{2}$	$1.8859\times 10^{2}$	$8.9728\times 10^{6}$	$1.3206\times 10^{7}$
ECO	$5.6526\times 10^{2}$	$2.6426\times 10^{1}$	$1.1725\times 10^{2}$	$4.4643\times 10^{1}$	$7.6896\times 10^{3}$	$3.7558\times 10^{3}$
PO	$5.4872\times 10^{2}$	$2.2812\times 10^{1}$	$5.4830\times 10^{1}$	$1.4481\times 10^{1}$	$4.3045\times 10^{3}$	$1.4688\times 10^{3}$
SBO	$5.3747\times 10^{2}$	$1.9882\times 10^{1}$	$4.2144\times 10^{1}$	$8.7300\times 10^{0}$	$3.3869\times 10^{3}$	$8.6362\times 10^{2}$
DE	$5.3454\times 10^{2}$	$2.7559\times 10^{1}$	$9.0802\times 10^{1}$	$6.6952\times 10^{1}$	$1.9382\times 10^{4}$	$3.8155\times 10^{4}$
PSO	$6.7781\times 10^{2}$	$1.0347\times 10^{2}$	$8.8366\times 10^{2}$	$3.3179\times 10^{2}$	$1.5493\times 10^{8}$	$2.2790\times 10^{8}$
GWO	$6.4208\times 10^{2}$	$2.0676\times 10^{1}$	$6.7049\times 10^{2}$	$3.9898\times 10^{1}$	$9.5966\times 10^{8}$	$3.5545\times 10^{8}$
	**F29**		**F30**			
**Algo.**	**Avg**	**Std**	**Avg**	**Std**		
GT-SBOA	$3.2064\times 10^{3}$	$2.1932\times 10^{3}$	$2.3116\times 10^{3}$	$1.4833\times 10^{3}$		
SBOA	$2.8443\times 10^{4}$	$1.8537\times 10^{4}$	$1.9332\times 10^{4}$	$1.2709\times 10^{4}$		
UTFSBOA	$1.4837\times 10^{4}$	$1.2103\times 10^{4}$	$7.0180\times 10^{3}$	$2.9007\times 10^{3}$		
ISBOA	$1.8077\times 10^{4}$	$1.1641\times 10^{4}$	$2.1252\times 10^{4}$	$1.4365\times 10^{4}$		
RIME	$5.0482\times 10^{4}$	$6.3889\times 10^{4}$	$2.0976\times 10^{5}$	$3.6783\times 10^{5}$		
ECO	$2.6838\times 10^{4}$	$1.6057\times 10^{4}$	$1.6226\times 10^{4}$	$1.8729\times 10^{4}$		
PO	$1.9426\times 10^{4}$	$2.2445\times 10^{4}$	$7.3453\times 10^{3}$	$3.2221\times 10^{3}$		
SBO	$9.1853\times 10^{3}$	$5.5166\times 10^{3}$	$4.4417\times 10^{3}$	$2.2597\times 10^{3}$		
DE	$1.5854\times 10^{4}$	$1.0910\times 10^{4}$	$5.0965\times 10^{3}$	$2.1734\times 10^{3}$		
PSO	$4.0043\times 10^{8}$	$6.3884\times 10^{8}$	$8.1018\times 10^{5}$	$8.5607\times 10^{5}$		
GWO	$1.7009\times 10^{9}$	$6.0519\times 10^{8}$	$4.0470\times 10^{8}$	$8.1764\times 10^{7}$		
**Overall Rank**						
**Algo.**	**Rank**	**Avg. Friedman Rank**	**Best Count**	**Mean W/T/L**	**Sig. W/T/L**	
GT-SBOA	1	2.4138	14	–	–	
UTFSBOA	2	3.9655	1	19/0/10	18/3/8	
ISBOA	3	4.0690	3	20/0/9	18/4/7	
SBOA	4	4.4138	9	16/0/13	16/4/9	
SBO	4	4.4138	1	25/0/4	20/8/1	
DE	6	5.3103	1	27/0/2	19/9/1	
PO	7	6.1034	0	27/0/2	24/5/0	
ECO	8	7.3793	0	29/0/0	26/3/0	
RIME	9	8.0000	0	28/0/1	24/4/1	
PSO	10	9.2759	0	29/0/0	29/0/0	
GWO	11	10.6552	0	29/0/0	29/0/0	

**Table 4:** Holm-adjusted, two-sided Wilcoxon rank-sum *p*-values for GT-SBOA versus the comparison algorithms on CEC2017 at D=30. Correction covers 29 functions separately for each comparator. Superscripts +/=/− denote significant wins, no detected difference and significant losses (α=0.05).

Function	SBOA	UTFSBOA	ISBOA	RIME	ECO	PO	SBO	DE	PSO	GWO
F1	${3.51\times 10^{-11}}^{+}$	${3.51\times 10^{-11}}^{+}$	${3.51\times 10^{-11}}^{+}$	${3.51\times 10^{-11}}^{+}$	${3.51\times 10^{-11}}^{+}$	${3.51\times 10^{-11}}^{+}$	${3.51\times 10^{-11}}^{+}$	${3.51\times 10^{-11}}^{+}$	${3.51\times 10^{-11}}^{+}$	${3.51\times 10^{-11}}^{+}$
F3	${6.55\times 10^{-8}}^{+}$	${2.08\times 10^{-7}}^{+}$	${8.22\times 10^{-4}}^{+}$	${8.00\times 10^{-9}}^{+}$	${1.17\times 10^{-6}}^{+}$	${3.07\times 10^{-6}}^{+}$	${6.85\times 10^{-6}}^{+}$	${1.24\times 10^{-6}}^{+}$	${8.46\times 10^{-10}}^{+}$	${8.46\times 10^{-10}}^{+}$
F4	${1.36\times 10^{-2}}^{-}$	${6.59\times 10^{-9}}^{+}$	${2.89\times 10^{-1}}^{=}$	${2.85\times 10^{-9}}^{+}$	${8.46\times 10^{-10}}^{+}$	${4.30\times 10^{-9}}^{+}$	${1.08\times 10^{-9}}^{+}$	${8.46\times 10^{-1}}^{=}$	${8.46\times 10^{-10}}^{+}$	${8.46\times 10^{-10}}^{+}$
F5	${8.55\times 10^{-8}}^{+}$	${3.45\times 10^{-9}}^{+}$	${1.03\times 10^{-9}}^{+}$	${8.46\times 10^{-10}}^{+}$	${8.46\times 10^{-10}}^{+}$	${8.46\times 10^{-10}}^{+}$	${1.63\times 10^{-9}}^{+}$	${1.77\times 10^{-6}}^{-}$	${1.66\times 10^{-9}}^{+}$	${8.46\times 10^{-10}}^{+}$
F6	${7.28\times 10^{-1}}^{=}$	${1.40\times 10^{-9}}^{+}$	${1.39\times 10^{-5}}^{+}$	${8.46\times 10^{-10}}^{+}$	${8.46\times 10^{-10}}^{+}$	${1.48\times 10^{-8}}^{+}$	${8.46\times 10^{-10}}^{+}$	${1.00\times 10^{0}}^{=}$	${8.46\times 10^{-10}}^{+}$	${8.46\times 10^{-10}}^{+}$
F7	${3.32\times 10^{-9}}^{+}$	${8.46\times 10^{-10}}^{+}$	${8.15\times 10^{-10}}^{+}$	${8.46\times 10^{-10}}^{+}$	${8.46\times 10^{-10}}^{+}$	${1.04\times 10^{-9}}^{+}$	${8.46\times 10^{-10}}^{+}$	${4.42\times 10^{-4}}^{+}$	${8.46\times 10^{-10}}^{+}$	${8.46\times 10^{-10}}^{+}$
F8	${3.51\times 10^{-11}}^{-}$	${8.46\times 10^{-10}}^{-}$	${8.85\times 10^{-11}}^{-}$	${8.46\times 10^{-10}}^{+}$	${8.46\times 10^{-10}}^{+}$	${1.11\times 10^{-7}}^{+}$	${8.46\times 10^{-10}}^{+}$	${9.96\times 10^{-4}}^{+}$	${1.66\times 10^{-9}}^{+}$	${8.46\times 10^{-10}}^{+}$
F9	${1.03\times 10^{-9}}^{+}$	${1.04\times 10^{-9}}^{+}$	${8.15\times 10^{-10}}^{+}$	${8.46\times 10^{-10}}^{+}$	${8.46\times 10^{-10}}^{+}$	${8.46\times 10^{-10}}^{+}$	${2.79\times 10^{-9}}^{+}$	${4.03\times 10^{-7}}^{+}$	${8.46\times 10^{-10}}^{+}$	${8.46\times 10^{-10}}^{+}$
F10	${8.15\times 10^{-10}}^{+}$	${3.50\times 10^{-8}}^{+}$	${1.03\times 10^{-9}}^{+}$	${8.46\times 10^{-10}}^{+}$	${5.35\times 10^{-8}}^{+}$	${4.81\times 10^{-8}}^{+}$	${1.01\times 10^{-4}}^{+}$	${8.79\times 10^{-9}}^{+}$	${8.46\times 10^{-10}}^{+}$	${8.46\times 10^{-10}}^{+}$
F11	${8.15\times 10^{-10}}^{+}$	${1.86\times 10^{-9}}^{+}$	${4.21\times 10^{-4}}^{+}$	${8.46\times 10^{-10}}^{+}$	${2.93\times 10^{-6}}^{+}$	${4.23\times 10^{-8}}^{+}$	${2.27\times 10^{-2}}^{+}$	${5.12\times 10^{-8}}^{+}$	${8.46\times 10^{-10}}^{+}$	${8.46\times 10^{-10}}^{+}$
F12	${1.13\times 10^{-6}}^{+}$	${3.37\times 10^{-5}}^{+}$	${1.35\times 10^{-3}}^{+}$	${1.69\times 10^{-9}}^{+}$	${6.68\times 10^{-5}}^{+}$	${9.40\times 10^{-4}}^{+}$	${8.98\times 10^{-2}}^{=}$	${2.25\times 10^{-3}}^{+}$	${8.46\times 10^{-10}}^{+}$	${8.46\times 10^{-10}}^{+}$
F13	${8.15\times 10^{-10}}^{+}$	${8.46\times 10^{-10}}^{+}$	${8.15\times 10^{-10}}^{+}$	${8.46\times 10^{-10}}^{+}$	${8.46\times 10^{-10}}^{+}$	${8.46\times 10^{-10}}^{+}$	${1.40\times 10^{-9}}^{+}$	${1.52\times 10^{-9}}^{+}$	${8.46\times 10^{-10}}^{+}$	${8.46\times 10^{-10}}^{+}$
F14	${8.15\times 10^{-10}}^{+}$	${3.45\times 10^{-9}}^{+}$	${2.26\times 10^{-9}}^{+}$	${8.46\times 10^{-10}}^{+}$	${8.46\times 10^{-10}}^{+}$	${6.63\times 10^{-9}}^{+}$	${2.80\times 10^{-6}}^{+}$	${2.89\times 10^{-9}}^{+}$	${8.46\times 10^{-10}}^{+}$	${8.46\times 10^{-10}}^{+}$
F15	${8.15\times 10^{-10}}^{-}$	${1.34\times 10^{-9}}^{-}$	${8.15\times 10^{-10}}^{-}$	${9.10\times 10^{-10}}^{+}$	${6.85\times 10^{-1}}^{=}$	${5.71\times 10^{-3}}^{+}$	${2.94\times 10^{-1}}^{=}$	${8.46\times 10^{-10}}^{+}$	${8.46\times 10^{-10}}^{+}$	${8.46\times 10^{-10}}^{+}$
F16	${8.15\times 10^{-10}}^{+}$	${1.18\times 10^{-7}}^{+}$	${2.24\times 10^{-5}}^{+}$	${4.62\times 10^{-9}}^{+}$	${3.66\times 10^{-8}}^{+}$	${2.20\times 10^{-5}}^{+}$	${2.27\times 10^{-2}}^{+}$	${1.00\times 10^{0}}^{=}$	${8.46\times 10^{-10}}^{+}$	${8.46\times 10^{-10}}^{+}$
F17	${8.15\times 10^{-10}}^{+}$	${3.45\times 10^{-9}}^{+}$	${8.15\times 10^{-10}}^{+}$	${8.46\times 10^{-10}}^{+}$	${8.46\times 10^{-10}}^{+}$	${8.46\times 10^{-10}}^{+}$	${3.16\times 10^{-4}}^{+}$	${1.29\times 10^{-9}}^{+}$	${8.46\times 10^{-10}}^{+}$	${8.46\times 10^{-10}}^{+}$
F18	${8.15\times 10^{-10}}^{+}$	${8.46\times 10^{-10}}^{+}$	${8.15\times 10^{-10}}^{+}$	${8.46\times 10^{-10}}^{+}$	${8.46\times 10^{-10}}^{+}$	${8.46\times 10^{-10}}^{+}$	${8.46\times 10^{-10}}^{+}$	${8.46\times 10^{-10}}^{+}$	${8.46\times 10^{-10}}^{+}$	${8.46\times 10^{-10}}^{+}$
F19	${1.08\times 10^{-1}}^{=}$	${7.02\times 10^{-1}}^{=}$	${2.89\times 10^{-1}}^{=}$	${2.54\times 10^{-2}}^{+}$	${1.47\times 10^{-6}}^{+}$	${9.83\times 10^{-1}}^{=}$	${2.04\times 10^{-2}}^{+}$	${1.00\times 10^{0}}^{=}$	${2.16\times 10^{-3}}^{+}$	${8.46\times 10^{-10}}^{+}$
F20	${1.43\times 10^{-7}}^{-}$	${8.36\times 10^{-6}}^{-}$	${2.89\times 10^{-1}}^{=}$	${1.26\times 10^{-1}}^{=}$	${2.97\times 10^{-8}}^{+}$	${9.40\times 10^{-4}}^{+}$	${4.21\times 10^{-1}}^{=}$	${5.34\times 10^{-2}}^{=}$	${8.46\times 10^{-10}}^{+}$	${8.46\times 10^{-10}}^{+}$
F21	${4.97\times 10^{-8}}^{-}$	${8.46\times 10^{-10}}^{-}$	${8.15\times 10^{-10}}^{-}$	${4.62\times 10^{-9}}^{-}$	${2.87\times 10^{-7}}^{+}$	${1.02\times 10^{-4}}^{+}$	${1.11\times 10^{-8}}^{-}$	${1.55\times 10^{-3}}^{+}$	${1.96\times 10^{-7}}^{+}$	${8.46\times 10^{-10}}^{+}$
F22	${2.32\times 10^{-1}}^{=}$	${1.00\times 10^{0}}^{=}$	${2.83\times 10^{-6}}^{+}$	${1.00\times 10^{0}}^{=}$	${6.85\times 10^{-1}}^{=}$	${9.83\times 10^{-1}}^{=}$	${1.00\times 10^{0}}^{=}$	${9.96\times 10^{-4}}^{+}$	${8.46\times 10^{-10}}^{+}$	${8.46\times 10^{-10}}^{+}$
F23	${3.98\times 10^{-2}}^{-}$	${1.00\times 10^{0}}^{=}$	${8.94\times 10^{-2}}^{=}$	${1.00\times 10^{0}}^{=}$	${6.85\times 10^{-1}}^{=}$	${1.00\times 10^{0}}^{=}$	${1.00\times 10^{0}}^{=}$	${4.95\times 10^{-5}}^{+}$	${8.46\times 10^{-10}}^{+}$	${8.46\times 10^{-10}}^{+}$
F24	${2.10\times 10^{-3}}^{-}$	${8.46\times 10^{-10}}^{-}$	${8.15\times 10^{-10}}^{-}$	${5.39\times 10^{-5}}^{+}$	${7.19\times 10^{-6}}^{+}$	${5.72\times 10^{-4}}^{+}$	${3.16\times 10^{-4}}^{+}$	${5.01\times 10^{-2}}^{=}$	${2.13\times 10^{-7}}^{+}$	${8.46\times 10^{-10}}^{+}$
F25	${2.32\times 10^{-1}}^{=}$	${8.46\times 10^{-10}}^{-}$	${8.15\times 10^{-10}}^{-}$	${1.61\times 10^{-1}}^{=}$	${1.43\times 10^{-7}}^{+}$	${2.07\times 10^{-5}}^{+}$	${1.00\times 10^{0}}^{=}$	${4.16\times 10^{-4}}^{+}$	${2.95\times 10^{-9}}^{+}$	${8.46\times 10^{-10}}^{+}$
F26	${4.84\times 10^{-6}}^{-}$	${1.79\times 10^{-9}}^{-}$	${8.15\times 10^{-10}}^{-}$	${1.11\times 10^{-3}}^{+}$	${5.91\times 10^{-9}}^{+}$	${5.47\times 10^{-7}}^{+}$	${1.75\times 10^{-5}}^{+}$	${8.55\times 10^{-2}}^{=}$	${1.91\times 10^{-9}}^{+}$	${8.46\times 10^{-10}}^{+}$
F27	${8.15\times 10^{-10}}^{-}$	${9.64\times 10^{-6}}^{-}$	${4.59\times 10^{-8}}^{-}$	${3.64\times 10^{-2}}^{+}$	${4.01\times 10^{-3}}^{+}$	${1.00\times 10^{0}}^{=}$	${1.00\times 10^{0}}^{=}$	${7.68\times 10^{-1}}^{=}$	${8.46\times 10^{-10}}^{+}$	${8.46\times 10^{-10}}^{+}$
F28	${1.35\times 10^{-9}}^{+}$	${2.48\times 10^{-3}}^{+}$	${1.35\times 10^{-3}}^{+}$	${8.46\times 10^{-10}}^{+}$	${2.85\times 10^{-5}}^{+}$	${3.74\times 10^{-1}}^{=}$	${1.00\times 10^{0}}^{=}$	${4.35\times 10^{-1}}^{=}$	${8.46\times 10^{-10}}^{+}$	${8.46\times 10^{-10}}^{+}$
F29	${1.30\times 10^{-9}}^{+}$	${8.91\times 10^{-9}}^{+}$	${1.53\times 10^{-8}}^{+}$	${8.46\times 10^{-10}}^{+}$	${8.46\times 10^{-10}}^{+}$	${5.35\times 10^{-6}}^{+}$	${2.22\times 10^{-5}}^{+}$	${3.10\times 10^{-8}}^{+}$	${8.46\times 10^{-10}}^{+}$	${8.46\times 10^{-10}}^{+}$
F30	${8.15\times 10^{-10}}^{+}$	${8.07\times 10^{-9}}^{+}$	${1.03\times 10^{-9}}^{+}$	${2.31\times 10^{-9}}^{+}$	${3.29\times 10^{-6}}^{+}$	${1.14\times 10^{-7}}^{+}$	${2.29\times 10^{-4}}^{+}$	${2.54\times 10^{-5}}^{+}$	${8.46\times 10^{-10}}^{+}$	${8.46\times 10^{-10}}^{+}$

**Table 5:** Across-function rank comparisons against GT-SBOA. Two-sided normal-approximation *p*-values are Holm-adjusted over all control comparisons within each dimension (10 at D=30, 3 at D=50 or 100).

*D*	Comparator	Mean Rank	Holm *p*
30	SBOA	4.4138	$8.66\times 10^{-2}$
30	UTFSBOA	3.9655	$1.15\times 10^{-1}$
30	ISBOA	4.0690	$1.15\times 10^{-1}$
30	RIME	8.0000	$1.14\times 10^{-9}$
30	ECO	7.3793	$8.34\times 10^{-8}$
30	PO	6.1034	$1.36\times 10^{-4}$
30	SBO	4.4138	$8.66\times 10^{-2}$
30	DE	5.3103	$4.41\times 10^{-3}$
30	PSO	9.2759	$2.98\times 10^{-14}$
30	GWO	10.6552	$3.02\times 10^{-20}$
50	SBOA	3.7414	$3.69\times 10^{-7}$
50	UTFSBOA	2.2586	$7.20\times 10^{-1}$
50	ISBOA	2.0517	$7.60\times 10^{-1}$
100	SBOA	3.0517	$3.09\times 10^{-1}$
100	UTFSBOA	2.4310	$6.84\times 10^{-1}$
100	ISBOA	1.9483	$2.01\times 10^{-1}$

**Table 6:** Component comparison at D=30. All methods use 30 runs, N=30 and *B* = 300,000. GT-SBOA and SBOA reuse exactly the records in Table 3. W/T/L is from the GT-SBOA perspective; significant counts use the same 29-test family as the main comparison.

Algorithm	Best	Mean Rank	Mean W/T/L	Sig. W/T/L
GT-SBOA	16	2.0345	–	–
T-SBOA	2	2.3448	21/0/8	17/4/8
GPS-SBOA	3	2.7931	20/0/9	18/4/7
SBOA	8	2.8276	16/0/13	16/4/9

**Table 7:** Directional-weight sensitivity at D=30: 29 functions and 30 records per setting. The active fraction is 0.5, and the alternative-branch probability is 0.35 throughout. The α=0.65 records are shared with Table 3.

α	Mean Rank
0.00	3.5690
0.35	4.1207
0.50	3.2586
0.65	3.0862
0.80	3.5000
1.00	3.4655

**Table 8:** Dimension-specific comparison. The ranks at D=30 use eleven algorithms; those at D=50 and 100 use four. Significant W/T/L is from the GT-SBOA perspective.

*D*	Algorithm	Mean Rank	Best	Sig. W/T/L
30	GT-SBOA	2.4138	14	–
30	SBOA	4.4138	9	16/4/9
30	UTFSBOA	3.9655	1	18/3/8
30	ISBOA	4.0690	3	18/4/7
50	GT-SBOA	1.9483	12	–
50	SBOA	3.7414	1	21/7/1
50	UTFSBOA	2.2586	9	10/13/6
50	ISBOA	2.0517	10	8/14/7
100	GT-SBOA	2.5690	6	–
100	SBOA	3.0517	2	14/10/5
100	UTFSBOA	2.4310	8	9/12/8
100	ISBOA	1.9483	16	6/9/14

**Table 9:** Independent-sample effect sizes for F3. $A_{\mathrm{lower}}>0.5$ favors GT-SBOA. Pointwise 95% percentile intervals use 2000 independent within-group bootstrap resamples; Holm *p* uses the same 29-function family as the corresponding benchmark comparison.

*D*	Comparator	$A_{\mathrm{lower}}$	95% Interval	Holm *p*
30	SBOA	0.939	[0.842, 1.000]	$6.55\times 10^{-8}$
30	UTFSBOA	0.919	[0.834, 0.979]	$2.08\times 10^{-7}$
30	ISBOA	0.790	[0.672, 0.897]	$8.22\times 10^{-4}$
50	SBOA	0.967	[0.900, 1.000]	$1.11\times 10^{-8}$
50	UTFSBOA	0.867	[0.733, 0.967]	$2.33\times 10^{-5}$
50	ISBOA	0.833	[0.700, 0.967]	$1.81\times 10^{-4}$
100	SBOA	0.831	[0.697, 0.934]	$1.64\times 10^{-4}$
100	UTFSBOA	0.837	[0.700, 0.967]	$1.39\times 10^{-4}$
100	ISBOA	0.667	[0.500, 0.833]	$2.17\times 10^{-1}$

**Table 10:** Algorithm update runtime in seconds at B=10,000D (mean ± sample standard deviation).

Algorithm	D=30	D=50	D=100
GT-SBOA	0.276±0.069	0.662±0.209	2.282±0.849
SBOA	0.295±0.093	0.669±0.222	2.222±0.744
UTFSBOA	0.333±0.082	0.771±0.269	2.861±0.994
ISBOA	0.378±0.109	0.839±0.285	3.267±1.196

**Table 11:** Common-initialization control against the parent SBOA. GT-SBOA (RI) uses the proposed search updates with random initialization replacing GPS. Both methods draw 30 independent uniform points in ${\left[0,1\right]}^{D}$ and apply the same repair, with no preliminary selection. Values are mean (sample standard deviation) over 30 complete frontiers. Superscripts +/=/− give significant wins, no detected difference and losses against SBOA using Wilcoxon rank-sum tests with Holm correction over 10 comparisons (5 datasets, 2 metrics).

Instance	Algorithm	GD	IGD
port1	GT-SBOA (RI)	$1.650\times 10^{-4}$ ($4.65\times 10^{-6}$)^+^	$2.241\times 10^{-2}$ ($3.98\times 10^{-7}$)^+^
	SBOA	$1.875\times 10^{-4}$ ($4.95\times 10^{-6}$)	$2.323\times 10^{-2}$ ($5.96\times 10^{-7}$)
port2	GT-SBOA (RI)	$6.988\times 10^{-4}$ ($1.49\times 10^{-5}$)^+^	$2.888\times 10^{-2}$ ($3.62\times 10^{-6}$)^+^
	SBOA	$7.219\times 10^{-4}$ ($2.04\times 10^{-5}$)	$3.306\times 10^{-2}$ ($4.80\times 10^{-6}$)
port3	GT-SBOA (RI)	$9.502\times 10^{-4}$ ($2.49\times 10^{-5}$)^+^	$2.303\times 10^{-2}$ ($1.05\times 10^{-6}$)^+^
	SBOA	$1.001\times 10^{-3}$ ($2.17\times 10^{-5}$)	$2.436\times 10^{-2}$ ($1.17\times 10^{-6}$)
port4	GT-SBOA (RI)	$1.117\times 10^{-3}$ ($2.74\times 10^{-5}$)^+^	$2.547\times 10^{-2}$ ($2.08\times 10^{-6}$)^+^
	SBOA	$1.220\times 10^{-3}$ ($3.65\times 10^{-5}$)	$2.771\times 10^{-2}$ ($3.98\times 10^{-6}$)
port5	GT-SBOA (RI)	$1.384\times 10^{-2}$ ($3.49\times 10^{-4}$)^−^	$3.194\times 10^{-2}$ ($1.40\times 10^{-4}$)^+^
	SBOA	$1.327\times 10^{-2}$ ($3.23\times 10^{-4}$)	$3.556\times 10^{-2}$ ($1.09\times 10^{-4}$)

**Table 12:** GD on five OR-Library instances: mean (sample standard deviation) over 30 independent complete frontiers, with 21 scalarizations per frontier. Lower is better. Best means are bold.

Algorithm	port1	port2	port3	port4	port5	Avg. Rank
GT-SBOA	$1.6335\times 10^{-4}$ ($4.84\times 10^{-6}$)	$6.9185\times 10^{-4}$ ($1.94\times 10^{-5}$)	$9.4080\times 10^{-4}$ ($2.28\times 10^{-5}$)	$1.1055\times 10^{-3}$ ($2.80\times 10^{-5}$)	$1.3700\times 10^{-2}$ ($4.21\times 10^{-4}$)	1.40
SBOA	$1.8747\times 10^{-4}$ ($4.95\times 10^{-6}$)	$7.2193\times 10^{-4}$ ($2.04\times 10^{-5}$)	$1.0007\times 10^{-3}$ ($2.17\times 10^{-5}$)	$1.2197\times 10^{-3}$ ($3.65\times 10^{-5}$)	$1.3265\times 10^{-2}$ ($3.23\times 10^{-4}$)	2.40
UTFSBOA	$6.0527\times 10^{-3}$ ($2.11\times 10^{-3}$)	$1.2130\times 10^{-2}$ ($4.67\times 10^{-3}$)	$8.7703\times 10^{-3}$ ($3.60\times 10^{-3}$)	$5.0680\times 10^{-3}$ ($1.36\times 10^{-3}$)	$1.8111\times 10^{-2}$ ($5.43\times 10^{-3}$)	5.80
ISBOA	$5.3383\times 10^{-3}$ ($1.96\times 10^{-3}$)	$8.5699\times 10^{-3}$ ($2.57\times 10^{-3}$)	$6.3612\times 10^{-3}$ ($2.32\times 10^{-3}$)	$6.0180\times 10^{-3}$ ($1.80\times 10^{-3}$)	$8.8226\times 10^{-3}$ ($2.50\times 10^{-3}$)	4.60
RIME	$1.7380\times 10^{-4}$ ($3.72\times 10^{-6}$)	$1.3128\times 10^{-3}$ ($3.74\times 10^{-5}$)	$1.6862\times 10^{-3}$ ($3.57\times 10^{-5}$)	$1.9241\times 10^{-3}$ ($5.55\times 10^{-5}$)	$2.5500\times 10^{-2}$ ($6.27\times 10^{-4}$)	3.20
ECO	$1.3120\times 10^{-3}$ ($3.08\times 10^{-5}$)	$1.5749\times 10^{-2}$ ($3.91\times 10^{-4}$)	$1.5956\times 10^{-1}$ ($3.80\times 10^{-3}$)	$2.1553\times 10^{-2}$ ($4.84\times 10^{-4}$)	$8.9310\times 10^{-2}$ ($2.52\times 10^{-3}$)	8.00
PO	$2.2540\times 10^{-2}$ ($6.75\times 10^{-4}$)	$3.4786\times 10^{-2}$ ($9.27\times 10^{-4}$)	$5.7883\times 10^{-2}$ ($1.50\times 10^{-3}$)	$3.3555\times 10^{-2}$ ($9.13\times 10^{-4}$)	$2.9008\times 10^{-1}$ ($7.26\times 10^{-3}$)	9.60
SBO	$1.8416\times 10^{-4}$ ($4.37\times 10^{-6}$)	$1.5846\times 10^{-3}$ ($4.88\times 10^{-5}$)	$3.0147\times 10^{-3}$ ($6.52\times 10^{-5}$)	$1.8924\times 10^{-2}$ ($5.28\times 10^{-4}$)	$6.9447\times 10^{-2}$ ($1.80\times 10^{-3}$)	4.80
DE	$8.9441\times 10^{-3}$ ($2.38\times 10^{-4}$)	$3.3220\times 10^{-2}$ ($7.69\times 10^{-4}$)	$5.8001\times 10^{-2}$ ($1.67\times 10^{-3}$)	$3.4293\times 10^{-2}$ ($7.96\times 10^{-4}$)	$2.8466\times 10^{-1}$ ($7.60\times 10^{-3}$)	9.40
PSO	$7.2266\times 10^{-2}$ ($1.97\times 10^{-3}$)	$5.1516\times 10^{-2}$ ($1.21\times 10^{-3}$)	$7.8189\times 10^{-2}$ ($1.45\times 10^{-3}$)	$3.0549\times 10^{-2}$ ($6.35\times 10^{-4}$)	$3.5571\times 10^{-1}$ ($7.69\times 10^{-3}$)	10.40
GWO	$3.2266\times 10^{-4}$ ($6.53\times 10^{-6}$)	$9.5127\times 10^{-3}$ ($2.24\times 10^{-4}$)	$9.2293\times 10^{-3}$ ($2.57\times 10^{-4}$)	$1.2101\times 10^{-2}$ ($2.64\times 10^{-4}$)	$1.1832\times 10^{-1}$ ($2.18\times 10^{-3}$)	6.40

**Table 13:** IGD on five OR-Library instances: mean (sample standard deviation) over 30 independent complete frontiers, with 21 scalarizations per frontier. Lower is better. Best means are bold.

Algorithm	port1	port2	port3	port4	port5	Avg. Rank
GT-SBOA	$2.241\times 10^{-2}$ ($4.67\times 10^{-7}$)	$2.888\times 10^{-2}$ ($3.93\times 10^{-6}$)	$2.303\times 10^{-2}$ ($9.40\times 10^{-7}$)	$2.547\times 10^{-2}$ ($2.03\times 10^{-6}$)	$3.188\times 10^{-2}$ ($1.68\times 10^{-4}$)	1.00
SBOA	$2.323\times 10^{-2}$ ($5.96\times 10^{-7}$)	$3.306\times 10^{-2}$ ($4.80\times 10^{-6}$)	$2.436\times 10^{-2}$ ($1.17\times 10^{-6}$)	$2.771\times 10^{-2}$ ($3.98\times 10^{-6}$)	$3.556\times 10^{-2}$ ($1.09\times 10^{-4}$)	3.20
UTFSBOA	$3.120\times 10^{-2}$ ($1.04\times 10^{-3}$)	$3.906\times 10^{-2}$ ($2.19\times 10^{-3}$)	$2.743\times 10^{-2}$ ($1.41\times 10^{-3}$)	$2.794\times 10^{-2}$ ($3.31\times 10^{-4}$)	$3.747\times 10^{-2}$ ($3.10\times 10^{-3}$)	6.40
ISBOA	$3.097\times 10^{-2}$ ($6.06\times 10^{-4}$)	$3.843\times 10^{-2}$ ($1.53\times 10^{-3}$)	$2.677\times 10^{-2}$ ($5.16\times 10^{-4}$)	$2.818\times 10^{-2}$ ($7.50\times 10^{-4}$)	$3.435\times 10^{-2}$ ($8.63\times 10^{-4}$)	5.60
RIME	$2.323\times 10^{-2}$ ($5.91\times 10^{-7}$)	$3.304\times 10^{-2}$ ($5.61\times 10^{-6}$)	$2.435\times 10^{-2}$ ($1.69\times 10^{-6}$)	$2.774\times 10^{-2}$ ($3.96\times 10^{-6}$)	$4.139\times 10^{-2}$ ($3.74\times 10^{-4}$)	3.80
ECO	$2.311\times 10^{-2}$ ($4.26\times 10^{-6}$)	$3.574\times 10^{-2}$ ($1.68\times 10^{-4}$)	$1.726\times 10^{-1}$ ($4.10\times 10^{-3}$)	$3.528\times 10^{-2}$ ($3.15\times 10^{-4}$)	$9.433\times 10^{-2}$ ($2.35\times 10^{-3}$)	6.80
PO	$3.389\times 10^{-2}$ ($6.71\times 10^{-4}$)	$4.882\times 10^{-2}$ ($8.16\times 10^{-4}$)	$6.489\times 10^{-2}$ ($1.53\times 10^{-3}$)	$4.441\times 10^{-2}$ ($7.69\times 10^{-4}$)	$2.904\times 10^{-1}$ ($7.19\times 10^{-3}$)	9.60
SBO	$2.323\times 10^{-2}$ ($5.00\times 10^{-7}$)	$3.303\times 10^{-2}$ ($4.67\times 10^{-6}$)	$2.439\times 10^{-2}$ ($5.32\times 10^{-6}$)	$3.360\times 10^{-2}$ ($3.17\times 10^{-4}$)	$7.609\times 10^{-2}$ ($1.62\times 10^{-3}$)	4.80
DE	$2.418\times 10^{-2}$ ($8.90\times 10^{-5}$)	$4.744\times 10^{-2}$ ($6.49\times 10^{-4}$)	$6.500\times 10^{-2}$ ($1.70\times 10^{-3}$)	$4.503\times 10^{-2}$ ($6.61\times 10^{-4}$)	$2.850\times 10^{-1}$ ($7.53\times 10^{-3}$)	9.00
PSO	$1.010\times 10^{-1}$ ($3.09\times 10^{-3}$)	$6.516\times 10^{-2}$ ($1.26\times 10^{-3}$)	$8.593\times 10^{-2}$ ($1.50\times 10^{-3}$)	$4.194\times 10^{-2}$ ($5.14\times 10^{-4}$)	$3.556\times 10^{-1}$ ($7.66\times 10^{-3}$)	10.40
GWO	$2.323\times 10^{-2}$ ($7.54\times 10^{-7}$)	$3.377\times 10^{-2}$ ($4.78\times 10^{-5}$)	$2.552\times 10^{-2}$ ($7.99\times 10^{-5}$)	$3.012\times 10^{-2}$ ($1.09\times 10^{-4}$)	$1.219\times 10^{-1}$ ($2.10\times 10^{-3}$)	5.40

**Table 14:** Risk-standard-deviation gap at matched expected return relative to the supplied efficient frontier, in basis points of the input return period; mean (sample standard deviation). Reference risk is linearly interpolated by return.

Algorithm	port1	port2	port3	port4	port5
GT-SBOA	0.043 (0.002)	0.524 (0.019)	0.394 (0.010)	1.023 (0.085)	7.056 (0.194)
SBOA	0.049 (0.002)	0.511 (0.016)	0.414 (0.012)	1.313 (0.066)	6.546 (0.138)
UTFSBOA	4.687 (1.565)	17.405 (5.574)	4.795 (2.082)	7.693 (2.062)	14.985 (3.806)
ISBOA	4.046 (1.326)	12.896 (3.730)	3.518 (1.112)	9.345 (2.435)	7.997 (2.046)

**Table 15:** Significant W/T/L for GT-SBOA over the five datasets. Independent-sample Wilcoxon rank-sum tests use one Holm family of 100 comparisons (5 datasets, 10 comparators, 2 metrics).

Comparator	GD	IGD
SBOA	4/0/1	5/0/0
UTFSBOA	5/0/0	5/0/0
ISBOA	4/0/1	5/0/0
RIME	5/0/0	5/0/0
ECO	5/0/0	5/0/0
PO	5/0/0	5/0/0
SBO	5/0/0	5/0/0
DE	5/0/0	5/0/0
PSO	5/0/0	5/0/0
GWO	5/0/0	5/0/0

## Data Availability

The numerical and experimental data used to support the findings of this study are included within the article.
